# Astrocytes deliver CK1 to neurons via extracellular vesicles in response to inflammation promoting the translation and amyloidogenic processing of APP

**DOI:** 10.1002/jev2.12035

**Published:** 2020-12-31

**Authors:** Zhigang Li, Mohammed Moniruzzaman, Raha M. Dastgheyb, Seung‐Wan Yoo, Meina Wang, Hongbo Hao, Jia Liu, Patrizia Casaccia, Carlos Nogueras‐Ortiz, Dimitrios Kapogiannis, Barbara S. Slusher, Norman J. Haughey

**Affiliations:** ^1^ Department of Neurology, Richard T. Johnson Division of Neuroimmunology and Neurological Infections Johns Hopkins University School of Medicine Baltimore Maryland USA; ^2^ Laboratory of Clinical Investigation National Institute on Aging Baltimore Maryland USA; ^3^ Advanced Science Research Center at the Graduate Center, Neuroscience Initiative City University of New York New York New York USA; ^4^ Johns Hopkins Drug Discovery Johns Hopkins University School of Medicine Baltimore Maryland USA

**Keywords:** amyloid beta, astrocytes‐neurons cross talk, extracellular vesicles, inflammation

## Abstract

Chronic inflammation is thought to contribute to the early pathogenesis of Alzheimer's disease (AD). However, the precise mechanism by which inflammatory cytokines promote the formation and deposition of Aβ remains unclear. Available data suggest that applications of inflammatory cytokines onto isolated neurons do not promote the formation of Aβ, suggesting an indirect mechanism of action. Based on evidence astrocyte derived extracellular vesicles (astrocyte derived EVs) regulate neuronal functions, and data that inflammatory cytokines can modify the molecular cargo of astrocyte derived EVs, we sought to determine if IL‐1β promotes the formation of Aβ indirectly through actions of astrocyte derived EVs on neurons. The production of Aβ was increased when neurons were exposed to astrocyte derived EVs shed in response to IL‐1β (astrocyte derived EV‐IL‐1β). The mechanism for this effect involved an enrichment of Casein kinase 1 (CK1) in astrocyte derived EV‐IL‐1β. This astrocyte derived CK1 was delivered to neurons where it formed a complex with neuronal APC and GSK3 to inhibit the β‐catenin degradation. Stabilized β‐catenin translocated to the nucleus and bound to *Hnrnpc* gene at promoter regions. An increased cellular concentration of hnRNP C promoted the translation of APP by outcompeting the translational repressor fragile X mental retardation protein (FMRP) bound to APP mRNA. An increased amount of APP protein became co‐localized with BACE1 in enlarged membrane microdomains concurrent with increased production of Aβ. These findings identify a mechanism whereby inflammation promotes the formation of Aβ through the actions of astrocyte derived EV‐IL‐1β on neurons.

## 1 INTRODUCTION

The neuropathogenesis of Alzheimer's disease (AD) begins decades before the onset of clinically measurable cognitive impairment (Sperling et al., 2011). Although the early pathogenic events that contribute to the development of AD are an intense area of investigation, there is a considerable amount of evidence that chronic inflammation precedes the onset of cognitive impairment in AD (Tan et al., 2007), and may contribute to the development of AD associated pathology. Indeed, a number of studies have reported elevated inflammatory markers in serum and cerebrospinal fluid (CSF) at the earliest stages of cognitive decline. For example, CSF levels of sTREM2 (suggestive of microglial activation) are elevated at the preclinical stage of subjective cognitive decline (Nordengen et al., 2019), and levels of the inflammatory cytokines interleukin (IL)‐1 receptor antagonist, IL‐1 converting enzyme, IL‐2, IL‐6, IL‐8, tumour necrosis factor alpha (TNFα), macrophage‐colony stimulating factor and transforming growth factor β1 are elevated in brain tissues from healthy subjects with normal cognition and a high AD pathological burden, compared to healthy individuals with normal cognition and low AD pathological burden (Wilberding et al., 2008). Similar increases of inflammatory cytokines and CD45^+^ lymphocytes have been detected in the CSF of patients with amnestic mild cognitive impairment at levels similar to those found in untreated patients with the neuroinflammatory conditions of clinically isolated syndrome and multiple sclerosis (Monson et al., 2014). In addition to the positive associations of inflammation with the onset of cognitive impairment, inflammation has also been associated with faster rates of cognitive decline. Heightened serum levels of the inflammatory proteins alpha(1)‐antichymotrypsin (Dik et al., 2005) IL‐1, C‐reactive protein (CRP) (Engelhart et al., 2004), and high baseline levels of TNFα are associated with accelerated rates of cognitive decline (Holmes et al., 2009), and findings from the analysis of >12,000 participants enrolled in the Atherosclerosis Risk in Communities Study found that an inflammation composite score or CRP levels in the top quartile during midlife was associated with a 7.8%–11.6% steeper rate of cognitive decline, compared to participants in the lowest quartile (Walker et al., 2019). Together these findings suggest that both systemic and brain inflammation contribute to the development of AD pathology and cognitive decline. However, a clear mechanistic link between inflammation and the development of AD pathology has been elusive.

While there is clear evidence that the over production and deposition of amyloid beta (Aβ) peptides in brain parenchyma begins during the earliest stages of AD concurrent with increased inflammatory markers (Beyreuther & Masters, 1991; Rovelet‐Lecrux et al., 2006; Scheuner et al., 1996), it is not clear whether systemic inflammation and/or neuroinflammation during this time directly contributes to the production and deposition of Aβ. Inflammation induced by the administration of lipopolysaccharide (LPS) into APP_swe_ transgenic mice increased the amount of intraneuronal APP 1.8‐fold, Aβ_1‐40/42_ three‐fold (Sheng, 2003), and reduced the microglial clearance of Aβ by mechanisms that involved the NLRP3 inflammasome (Tejera et al., 2019), and altered TLR4 signalling (Go et al., 2016). However, the mechanism by which inflammation drives Aβ production is not clear. Available experimental evidence suggests that inflammatory cytokines do not directly act on neurons to promote the formation of Aβ peptides. Exposing cell lines and primary neurons in culture to inflammatory cytokines can increase the production of APP, but does not increase the production of Aβ peptides (Goetzl et al., 2019; Rogers et al., 1999), with the exception of a single study that demonstrated a combination of TNFα and IFNγ triggers the production of Aβ peptides in Sk‐n‐sh neuronal cells and thyroid epithelial cells (Blasko et al., 1999). These findings highlight a significant gap in our knowledge of how a chronic inflammatory state contributes to the development of Aβ brain pathology.

The lack of evidence for a direct effect of inflammatory cytokines on Aβ production in neurons suggests the involvement of an indirect mechanism. Based on accumulating evidence that extracellular vesicles (EVs) play a role in the pathogenesis of AD, we hypothesized that EVs shed from astrocytes in response to inflammatory cytokines promote Aβ production in neurons. Several lines of evidence suggests that EVs may play a role in the pathogenesis of AD. Neural precursor derived EVs isolated from the blood of patients with pre‐symptomatic AD contain reduced amounts of growth factors (Goetzl et al., 2019) compared to healthy control subjects. Likewise, peripheral blood EVs enriched for neuronal origin from pre‐symptomatic AD patients carry increased amounts of AD pathogenic proteins including P‐S396‐tau, P‐T181‐tau, APP, Aβ_1‐42_ (Fiandaca et al., 2015; Kapogiannis et al., 2019; Rajendran et al., 2006; Sharples et al., 2008), insulin receptor substrate‐1 (Kapogiannis et al., 2019), lysosomal proteins (Goetzl et al., 2015), neuronal survival factors (Goetzl et al., 2015) and pre‐synaptic proteins (Goetzl et al., 2018) compared cognitively normal controls. Blood derived EVs enriched for astrocyte markers contain elevated amounts of BACE1, sAPPβ, C1q, C4b, C3d, factor B, factor D, Bb, C3b and the C5b‐C9 terminal complement complex, and reduced amounts of glial derived neurotrophic factors in patients with AD compared with controls (Goetzl et al., 2016, Goetzl et al., 2018), suggesting innate immune activation with reduced neuronal support factors. These biomarkers change presumably reflect pre‐ and symptomatic AD pathology, but also raise the possibility that EVs may themselves play a mechanistic role in the development of AD pathology. Indeed, EVs have been implicated in the spread of amyloid and Tau pathology in pre‐clinical model systems (Asai et al., 2015; Baker et al., 2016; Crotti et al., 2019; Dinkins et al., 2016; Rajendran et al., 2006; Sardar Sinha et al., 2018), and attenuating the release of EVs by inhibition of neutral sphingomyelinase2 (nSMase2) has been shown to reduce Aβ plaque load in the 5XFAD mouse model (Dinkins et al., 2016). These data suggest that extracellular vesicles may play a role in the brain's response to inflammation in AD.

Here we explored the possibility that astrocyte derived EVs shed in response to the inflammatory cytokine IL‐1β are delivered to neurons where their cargo promotes the production of Aβ. There is a considerable amount of evidence that astrocyte derived EVs play an important role in regulating neuronal functions through the delivery of protein and RNA cargo to recipient neurons (Chaudhuri et al., 2018; Datta Chaudhuri et al., 2020; Fröhlich et al., 2014; Frühbeis et al., 2012, Frühbeis et al., 2013; Gosselin et al., 2013; Guitart et al., 2016; Hu et al., 2012; Lewis, 2013; Wang et al., 2011; Xin et al., 2017). Moreover, the molecular cargo of astrocyte derived EVs can be modified by the type of stimulus used to evoke their release (Chaudhuri et al., 2018; Datta Chaudhuri et al., 2020; Sami Saribas et al., 2017; Taylor et al., 2007). Based on these data we sought to determine if the inflammatory cytokine IL‐1β enhances Aβ formation indirectly through actions of astrocyte derived EVs on neurons.

## 2 MATERIALS AND METHODS

### 2.1 Primary neuron isolation and culture

Primary cortical and hippocampal neurons were isolated from day 18 embryos of Sprague‐Dawley rats as described previously (Bae et al., 2014; Chaudhuri et al., 2018). In brief, cortical and hippocampal tissues were separately dissociated by gentle trituration in a calcium‐free Hank's balanced salt solution and centrifuged at 1000 × *g*. Cells were resuspended in Neurobasal media (Gibco) containing 1% B27 supplement (Thermo Fisher Scientific), 1% antibiotic/antimitotic solution (10^4^ unit of penicillin G/ml, 10 mg streptomycin/ml and 25 μg amphotericin B/ml) (Sigma). Cortical neurons were plated at a density of 500,000 cells/ml in 6‐well plates, and hippocampal neurons were plated at a density of 150,000 cells/ml in 12 well plates with polyethyleneimine (Sigma) coated coverslips. Three hours after cell plating the medium was replaced with serum‐free Neurobasal medium containing 1% B27 supplement (Gibco). Routine immunofluorescent staining showed that cultures were >98% MAP‐2+ neurons. Cortical cultures were used between 7 and 10 days, and hippocampal cultures were used between 14 and 21 days in vitro. All animal procedures were approved by the Johns Hopkins University Animal Care and Use Committee.

### 2.2 Astrocyte isolation and culture

Primary rat cortical astrocyte cell cultures were established and maintained as described previously (Haughey & Mattson, 2003). Cortex of postnatal day 1 Sprague‐Dawley rats was mechanically dissociated in Hanks’ balanced salt solution, and plated on poly‐d‐lysine (Sigma) coated T175 culture flasks (Corning) containing Dulbecco's modified Eagle's medium/F‐12 media (Gibco BRL) supplemented with 10% fetal bovine serum (Gibco BRL), d‐glucose (final concentration 25 mM; Sigma), and 1% antibiotic/antimitotic solution (10^4^ Unit of penicillin G/ml, 10 mg streptomycin/ml and 25 μg amphotericin B/ml; Sigma). Type 1 astrocytes were purified by mechanical disassociation of less adherent cells as previously reported (Dickens et al., 2017). Culture flasks were secured to a rotary shaker (Thermo Scientific) and rotated at 200 rpm for 18 h at 37°C in a 5% CO_2_ tissue culture incubator. Less adherent cells were removed with the media, and fresh media was added to the cells. Cells were split when close to confluent and at passage 2 cultures were 98% GFAP+ astrocytes with type I morphology. Astrocytes were used for experiments between passages 3 and 10.

### 2.3 Astrocyte‐derived extracellular vesicles isolation

Culture rat primary astrocytes were grown to 70%–80% confluency. Astrocytes culture medium were removed, and fresh pre‐warmed serum‐free DMDM/F12 culture medium (supplemented with 25 mM d‐glucose and 1% antibiotic/antimitotic solution) was added. Cultures were treated with IL‐1β (200 ng/ml; R&D Systems) for 2 h, culture medium was collected, and astrocyte derived EVs were isolated as described previously (Dickens et al., 2017). Culture media was centrifuged at 3000 × *g* for 15 min at 4°C to remove cellular debris. The supernatant was collected and ultra‐centrifuged at 10,000 × *g* for 30 min at 4°C to remove larger microvesicles and apoptotic bodies. The resulting supernatant was ultra‐centrifuged at 100,000 × *g* for 3 h at 4°C to obtain the astrocyte derived EV pellet. For each EV isolation 4 T‐150 flasks of astrocytes (70%–80% confluency) were used to isolated EVs for a single experimental condition. The EV pellet was re‐suspended in 200 μl PBS for further analysis or experiments. In all experiments neurons were treated with a concentration of 50 astrocyte derived EVs per cell.

### 2.4 Nanoparticle tracking analysis

The size and number of astrocyte derived EVs were quantified using a ZetaView Nanoparticle Tracker (Particle Metrix GmBH, Meerbusch, Germany), and corresponding ZetaVeiw software (8.03.04.01). The instrument was calibrated prior to each use with 100 nm diameter beads (Thermo Scientific). Instrument pre‐acquisition parameters were set to a temperature of 23°C, sensitivity of 80, frame rate of 30 frames per second (fps), shutter speed of 100, and a laser pulse duration equal to that of shutter duration. Post‐acquisition parameters were set to a minimum brightness of 25, maximum size of 200 pixels, and a minimum size of 10 pixels. For each sample, 1 ml of diluted EVs were injected into the sample‐carrier cell and the particle count was measured at five positions, with two cycles of reading per position. The sample‐carrier cell was washed with PBS after every sample.

### 2.5 Western blot

Primary neuron, astrocyte or astrocyte derived EV pellets were lysed using RIPA buffer (50 mM Tris‐Cl, pH 7.5, 150 mM NaCl, 10 mM EDTA, 2 mM EGTA, 50 mM NaF, 0.5% SDS, 1% NP‐40) with the protease inhibitor cocktail (cOmplete, Sigma‐Aldrich) and phosphatase inhibitor (phosphoSTOP, Sigma‐Aldrich). Protein concentrations were determined using the BCA protein assay reagent kit (Thermo Fisher Scientific). Equal amount of proteins was loaded into each lane, and proteins were separated by SDS‐PAGE, then transferred to nitrocellulose membranes using iBlot 2 Dry Blotting System (Thermo Fisher Scientific). After blocking for 1 h at room temperature with 5% nonfat milk (Bio‐rad), the membranes were incubated overnight at 4°C with primary antibodies in 5% nonfat milk. A full list of primary antibodies can be found in Table S3. Membranes were washed in TBST (TBS contain 1% Tween‐20) and exposed to the appropriate horseradish peroxidase‐conjugated secondary antibody (1:2000, Cell Signaling Technology) for 1 h. Immunoreactive proteins were visualized by chemiluminescence (Millipore) using a QBOX imaging system (Syngene), and quantified using ImageJ version 1.50i.

### 2.6 Amyloid β_1‐42_ ELISA

Human amyloid‐β_1‐42_ was measured in culture supernatants and cell lysates according to the manufacturer's instructions (ThermoFisher Scientific; kit KHB3441). Briefly, 50 ml sample was loaded into the supplied 96 well plate and to this 50 ml of Aβ_42_ detection antibody solution was added. The mixture was incubated for 3 h at room temperature with gentle shaking. Wells were then washed 4 times with the supplied 1X wash buffer and 100 ml anti‐rabbit IgG HRP was added into each well, and the mixture incubated at room temperature for 30 min. Plates were then washed four times with 1X wash buffer and 100 ml stabilized chromogen added into each well and incubated for 30 min at room temperature. Stop solution was added to each well and read on a fluorescent plate reader at 450 nm absorbance (SpectraMax M2; Molecular Devices). Resulting data were fitted to a standard curve using SoftMax Pro Software.

### 2.7 Immunoprecipitation

Dynabeads (2 × 10^7^ M‐450 Epoxy; Thermo Fisher Scientific) were incubated with 2 μg primary antibody for each condition in a tube rotator overnight at 4°C. The antibody coupled beads were then incubated with cell lysate in a tube rotator overnight at 4°C. Immunobeads were washed three times with PBS, and resuspended by 1X sample buffer and boiled at 98°C for 5 min to denature the proteins. Immunoprecipitated proteins were detected by Western blot.

### 2.8 Quantitative RT‐qPCR

Total RNA was isolated from primary cortical cells using the RNeasy Mini Kit (Qiagen). Plasmid was synthesized using total RNA, N6 random primers and SuperScriptII Reverse Transcriptase (Invitrogen). Plasmid was then mixed with RNase free water, gene‐specific primers, and 2× PCR universal master mix (Applied Biosystems), RNA was amplified using the ABI 7500 Real Time PCR system. The gene specific primers used in this study are listed in Table S1. Fold change was calculated using the ΔΔ threshold cycle method, and data normalized to control groups.

### 2.9 Protein‐RNA immunoprecipitation and RT‐qPCR

To prepare the Dynabeads‐antibody complex, a primary antibody direct against hnRNP C (2 μg antibody, Santa Cruz Biotechnology) was incubated with Dynabeads (50 μl, Thermo Fisher Scientific) overnight at 4°C. Following experimental treatments, cells were lysed in RIPA buffer, disrupted by sonication, and centrifuged at 10,000 × *g* for 15 min at 4°C. Cell homogenates were collected and incubated with hnRNP C Ab‐Dynabead complex at 4°C overnight to obtain the Dynabead‐Protein‐RNA complex. The Dynabead‐Protein‐RNA complex was washed with wash PBS supplemented with 0.1% bovine serum albumin (BSA) and 2 mM EDTA (pH 7.4). Total RNA was isolated using TRIzol RNA Isolation Reagents (Thermo Fisher Scientific) following manufacturer's instructions. For quantitative analysis of mRNA binding with hnRNP C protein, RNA was isolated using TRIzol (Invitrogen), reverse‐transcribed using random hexamers and SSII reverse transcriptase (Invitrogen), and assayed for abundance of transcripts by RT‐qPCR analysis using SYBR Green PCR master mix (Applied Biosystems). The gene‐specific primers used for amplifying *APP* genes are listed in Table S1. Fold change was calculated using the ΔΔ threshold cycle method, which was normalized to control groups.

### 2.10 Chromatin immunoprecipitation ChIP‐qPCR analysis

ChIP was performed as previously described (Liu et al., 2015). Briefly, chromatin was isolated from rat primary cortical neurons treated with astrocyte derived EV‐CR, astrocyte derived EV‐IL‐1β, astrocyte derived EV‐siCK1‐IL‐1β and Wnt agonist I (19903, Cayman Chemical) for 18 h. DNA was sheared with Bioruptor Pico (Diagenode) and the size of the DNA in the sheared chromatin fragments was tested prior to precipitation to ensure the majority of fragment size was 200–400 bp. Immunoprecipitation was performed with *Staphylococcus aureus* protein A (Staph A)‐positive cells following ChIP protocols published on Peggy Farnham's laboratory website with minor modifications. Two micrograms of anti‐β‐catenin antibodies (Thermofisher 71–2700, Lot UB280762) were used per 1 unit OD260 readings. Rabbit IgG was used as a negative control. The DNA recovered from chromatin which was not immunoprecipitated was used as input. A detailed copy of the protocol can be found at http://farnham.genomecenter.ucdavis.edu/pdf/FarnhamLabChIP%20Protocol.pdf. The primer sequences for ChIP‐qPCR are given in Table S2. To identify potential β‐catenin binding site, sequences from ‐3 kb to +0.5 kb from the transcription start site of *Hnrnpc* gene were extracted from UCSC rn6 build and inputted into Genomatix. Potential binding sites were predicted using MatInspector function (Cartharius et al., 2005) provided by Genomatix.

### 2.11 Transfection and knockdown

Cultured astrocytes at 60%‑‐80% confluence were transfected between passages 3 and 6 following the manufacturer's instructions. For Myc‐Tag CK1 overexpression, 20 μg Myc‐Tag CK1 plasmid (Csnk1a1, Origene) or Myc‐Tag Vector plasmid (pCMV6‐Entry, Origene) was transfected into primary astrocytes using lipofectamine 3000. The plasmid‐lipid complexes incubated with astrocytes in T‐150 flask for 2 days at 37°C. Western blots were used to detect Myc‐Tag expression to confirm the effectiveness of the Myc‐Tag CK1 overexpression. For CK1 knockdown, 15 and 30 nM siCK1 (CSNK1A1, rn.Ri.Csnk1a1.13.1, Integrated DNA Technologies) and siNC (negative control DsiRNA, Integrated DNA Technologies) in lipofectamine RNAiMAX reagent. The siRNA‐lipid complexes incubated with astrocytes for 2 days at 37°C. Western blots were used to detect CK1 expression to confirm the effectiveness of the CK1 knockdown.

### 2.12 Immunofluorescence stain

For immunofluorescence experiments, primary hippocampal neurons were cultured on glass coverslips. Cholera toxin subunit B conjugated with AlexaFluor 555 (binds the ganglioside GM1; CTB‐555; Invitrogen) was used to identify lipid raft membrane microdomains. CTB‐555 (1 ng/ml) was incubated with neurons for 10 min at 37°C in a 5% CO_2_ incubator. Media was rapidly removed and cells were fixed with ice‐cold 4% paraformaldehyde in PBS. After washing with PBS, cells were permeabilized with 0.01% Triton X‐100 in PBS for 15 min at room temperature, followed by blocking with 5% horse serum and 5% goat serum in PBS for 1 h at room temperature. The cells were then incubated with primary antibody (Table S3) at 4°C overnight, followed by incubation with fluorescence‐conjugated secondary antibody, including anti‐rabbit AlexaFluor 488, anti‐mouse Cy5, anti‐mouse AlexaFluor 488 for 1 h at room temperature. Cell nuclei were stained with Hoechst 33258 (Sigma‐Aldrich), and cells imaged using a Zeiss inverted fluorescence microscope and Zen software (Carl Zeiss Inc.). Z‐Stack images were created using 12 slices with an interval of 0.27μm so that the total Z‐stack was 2.97 μm thick. Orthogonal images were created using Zen software. APP‐BACE1‐GM1 co‐localization was quantified by calculating the percentage of axonal area that showed co‐localization of all three fluorophores (Matlab 2018a). GSK3‐Myc, APC‐Myc and Axin1‐Myc co‐localization was quantified by Pearson's coefficient in ImageJ. Fluorescence intensity was calculated by ImageJ.

### 2.13 Human plasma samples

Serum samples were obtained from 4 patients with probable AD as determined by CSF biomarkers including low Aβ_42_, high Tau, and high p181‐Tau. An equal number of age‐ and sex‐matched cognitively normal subjects were included as controls (Table 1). Informed consent was obtained from each subject, and experimental procedures were approval by the institutional review board of the National Institutes of Health.

**TABLE 1:** Normal individuals and Alzheimer's disease patients’ information

	Age	Gender		Age	Gender	Clinic Stage	CSF Aβ (ng/l)	CSF tau (ng/l)	CSF p‐tau (ng/l)
Normal‐1	62	Female	**AD‐1**	60	Female	Multidomain MCI	200	52	56
Normal‐2	75	Female	**AD‐2**	72	Female	Mild Dementia	186	53	22
Normal‐3	81	Female	**AD‐3**	80	Female	Multidomain MCI	134	71	59
Normal‐4	83	Male	**AD‐4**	83	Male	Mild Dementia	141	61	46

Table graphs showing the information of Alzheimer's disease patients’ age, gender, clinical stage, and concentration of CSF Aβ, tau, p‐tau as well as the age‐ and gender‐matched normal individuals.

### 2.14 GLAST‐1^+^ extracellular vesicle isolation

Plasma (200 μl) was first centrifuged at 3000 × *g* for 20 min at 4°C to remove any debris. Plasma was then incubated with 126 μl of ExoQuick exosome solution (EXOQ; System Biosciences, Inc., Mountain View, CA, USA) for 60 min at room temperature to precipitate total EVs as described (Goetzl et al., 2015). EV pellets were resuspended in 350 μl of PBS for further analysis. To enrich for astrocyte derived EVs, the suspensions were incubated for 1 h at 4°C with 1.5 μg of mouse anti‐human glutamine aspartate transporter (GLAST) (ACSA‐1) biotinylated antibody (Miltenyi Biotec, Inc., Auburn, CA, USA) with 50 μl of 3% BSA, followed by adding 10 μl of streptavidin‐agarose UltraLink Resin (Thermo Fisher Scientific) in 40 μl of 3% BSA and incubated on a tube rotor for 30 min at 4°C. Tubes were centrifuged at 400 × *g* for 10 min at 4°C and the supernatant discarded. The pellet was resuspended in 100 μl of 0.05 M glycine‐HCl (pH 3.0) by gentle mixing for 10 s, and centrifuged at 4000 × *g* for 1 min at 4°C to elute the astrocyte derived EVs from resin. Astrocyte derived EVs were counted by nanoparticle tracking system (NanoSight). For immunoblotting, the protein cargo of astrocyte derived EVs was isolated by adding 4 μl of mammalian protein extraction reagent (M‐PER; Thermo Fisher Scientific) containing a cocktail of protease and phosphatase inhibitors to 1 × 10^9^ astrocyte derived EVs. The resulting suspensions were incubated at room temperature for 10 min, then 5X sample buffer was added and the mixture was boiled at 98°C for 5 min for western blot analysis. The size and concentration of EVs were measured using the nanoparticle tracking analysis system (NanoSight, Figure S5A).

### 2.15 Lipid extraction and LC‐ESI‐MS/MS analysis

Lipid extractions of cell samples were performed using a modified Bligh&Dyer (Bligh & Dyer, 1959) method. Briefly, cell pellets were lysed and homogenized in water followed by addition of methanol containing internal standards ceramide (d18:1/12:0) and sphingomyelin (d18:1/12:0) (Avanti Polar Lipids, Alabaster, AL, USA) (Haughey et al., 2004) at a concentration of 17 ng/ml. After the clear phase separation, organic layers containing crude lipid extracts were collected and dried in a nitrogen evaporator (Organomation, Berlin, MA, USA) and stored at ‐80°C. Dried extracts were resuspended in pure methanol prior to analysis.

### 2.16 Ceramide and sphingomyelin analysis

Chromatographic separations of ceramide and sphingomyelin present in the cell samples were performed on a C18 reverse‐phase column (2.6 μm, 50 × 2.1 mm) with an ULTRA HPLC In‐Line Filter (0.5 μm Depth Filter x 0.004 in ID) (Phenomenex, Torrance, CA, USA) using a Shimadzu ultra‐fast liquid chromatography system (Shimadzu, Nakagyo‐ku, Kyoto, Japan) coupled to a hybrid triple quadrupole LIT (linear ion trap) mass spectrometer 4000 QTRAP system equipped with Turbo Ion Spray (SCIEX, Foster City, CA, USA). Electrospray Ionization (ESI, +ve) was used to ionize these lipid species and individual ceramide and sphingomyelin species were detected by multiple reaction monitoring. Instrument conditions and HPLC parameters were similar to those described in previous studies (Bandaru et al., 2007; Mielke et al., 2015). In order to monitor the instrument condition over the run of samples, quality control (QC) samples were injected in every 10 injections. Eight‐point calibration curves (0.1–1000 ng/ml) were constructed by plotting area under the curve for each ceramide calibration standard d18:1/C16:0, d18:1/C18:0, d18:1/C20:0, d18:1/C22:0, d18:1/C24:0 and SM calibrations standards C16:0, C18:0, C20:0, C22:0 and C24:0 (Avanti Polar Lipids, Alabaster, AL, USA). Correlation coefficients for standard curves were >0.999. Ceramide and SM concentrations were calculated by fitting the identified ceramide and SM species to these standard curves based on acyl chain length. Instrument control and data acquisition were performed by using Analyst (version 1.4.2; SCIEX Inc., Thornhill, Ontario, Canada) and data analysis were completed using MultiQuant software (version 2.0, SCIEX).

### 2.17 Statistical analysis

The data are presented as mean ± SEM. GraphPad Prism v.8.2.0 was used for statistical analysis. Group differences were determined by ANOVA with Tukey post hoc comparisons unless otherwise specified, and *P* value < 0.05 was considered as statistically significant.

## 3 RESULTS

### 3.1 IL‐1β up‐regulates the release of astrocyte derived extracellular vesicles

**FIGURE 1:**
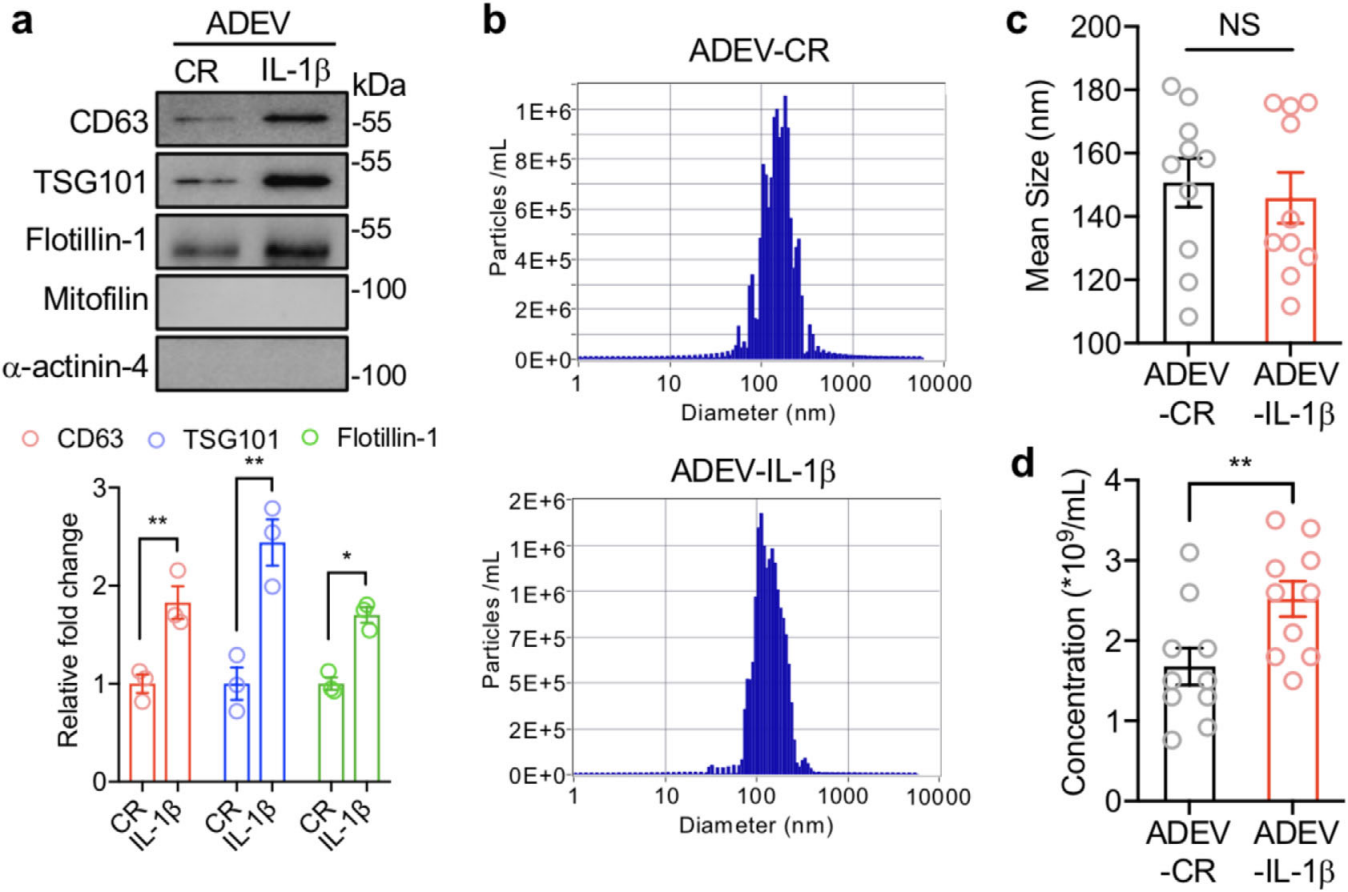
IL‐1β increases the release of astrocyte derived extracellular vesicles. Astrocyte derived extracellular vesicles shed in response to IL‐1β (200 ng/ml; astrocyte derived EV‐IL‐1β as ADEV‐IL‐1β) or constitutively released (astrocyte derived EV‐CR as ADEV‐CR) were isolated from media using a multi‐step ultracentrifugation. (a) Isolated astrocyte derived EVs were immunobloted for the tetraspanin protein CD63, The ESCRT protein TSG101, the lipid raft protein flotillin‐1, the mitochondrial protein mitofilin, and the cytoskeletal protein a‐actinin‐4. Data are mean ± SEM of *n* = 3 independent experiments per condition. *P < 0.05, **P < 0.01, Student's t‐test. (b) Representative histograms showing the size distribution and number of particles per millilitre using samples isolated from astrocyte derived EV‐CR and astrocyte derived EV‐IL‐1β. The (c) size and (d) concentration of astrocyte derived EVs isolated from the indicated experimental conditions are shown. Data are mean ± SEM of *n* = 10 independent experiments per condition. **P < 0.01. Student's t‐test

We focused our efforts on IL‐1β, as this inflammatory cytokine is elevated during the prodromal stages of AD (King et al., 2018), and is an initiating cytokine in pro‐inflammatory cytokine cascades. EVs shed from astrocytes in response to IL‐1β (200 ng/ml; astrocyte derived EV‐IL‐1β, ADEV‐IL‐1β) or constitutively released (CR) EVs (astrocyte derived EV‐CR, ADEV‐CR) were isolated from culture media using a multi‐step ultracentrifugation procedure (Dickens et al., 2017). Isolated astrocyte derived EVs were enriched for the tetraspanin protein CD63, the ESCRT protein TSG101, the lipid raft protein Flotillin‐1, and did not show any immunoreactivity for antibodies specific for the mitochondrial protein mitofilin, and the cytoskeletal protein α‐actinin‐4 (positive and negative EV markers by MISEV criteria (Théry et al., 2018)) (Figure 1a). The average size of astrocyte derived EV‐CR (150.68 ± 7.706 nm), and astrocyte derived EV‐IL‐1β (145.88 ± 7.995 nm) were similar (Figures 1b and 1c). However, there was a 47% increase in the number of astrocyte derived EV‐IL‐1β (2.52 ± 0.22 × 10^9^/ml), compared with astrocyte derived EV‐CR (1.678 ± 0.229 × 10^9^/ml) (Figures 1b and 1d). These data demonstrate that IL‐1β accelerates the release of EVs from astrocytes.

### 3.2 Astrocyte derived EV‐IL‐1β promotes APP translation through hnRNP C

**FIGURE 2:**
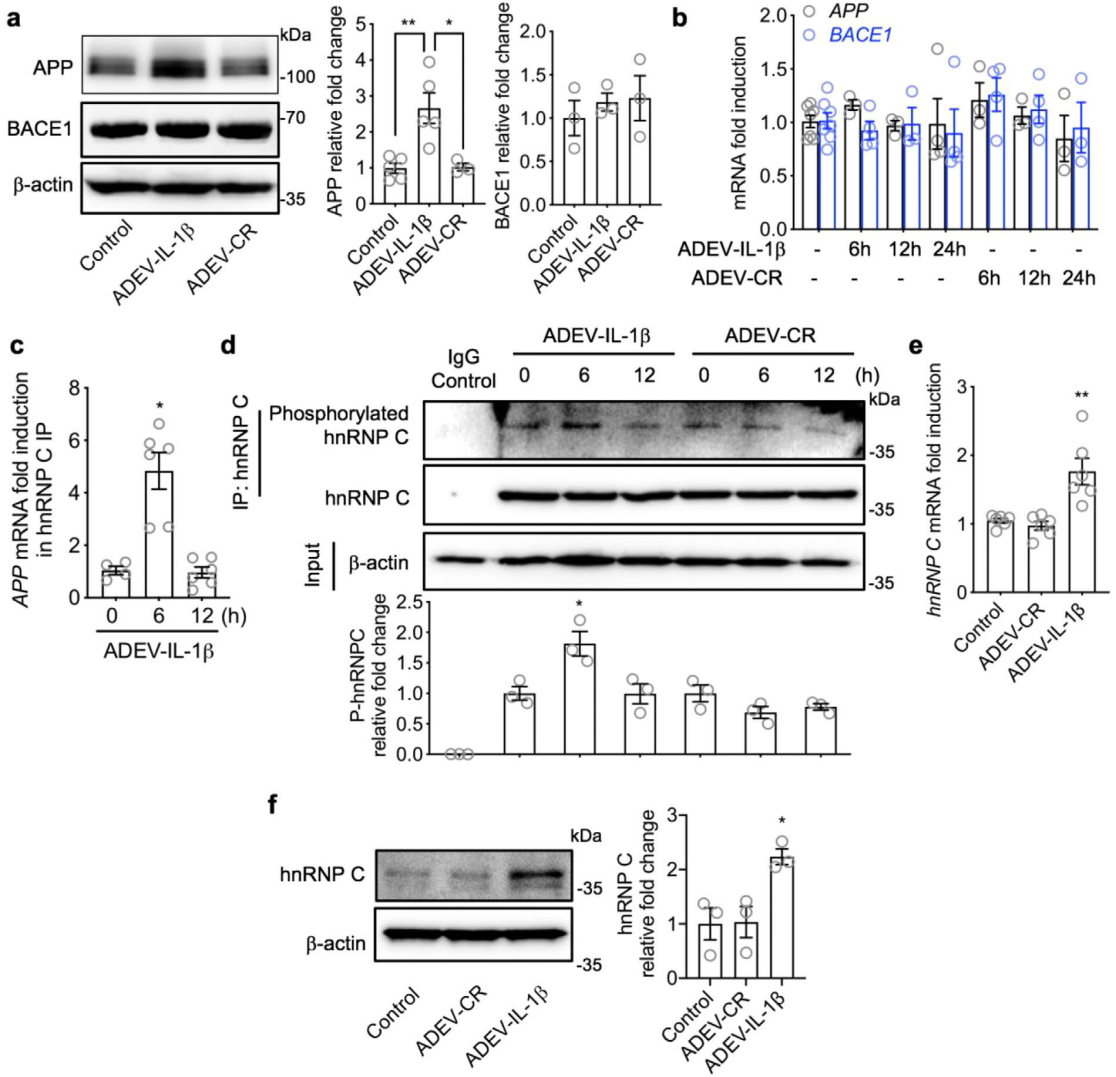
Astrocyte derived EV‐IL‐1β promotes APP translation through hnRNP C. Primary neurons were treated with vehicle (control), astrocyte derived EV‐CR (ADEV‐CR) or astrocyte derived EV‐IL‐1β (ADEV‐IL‐1β) (50 EVs per neuron) for 6–24 h as indicated. (a) Representative Western blot showing APP and BACE1 detected from neuronal lysates 24 h following the indicated treatment conditions. Bar graphs show densitometric quantitation of APP and BACE1 following the indicated treatments and time points. Data are mean ± SEM of *n* = 3 independent experiments per condition. *P < 0.05, **P < 0.01, one‐way ANOVA with Tukey post‐hoc comparisons. (b) Bar graphs show quantitative RT‐qPCR of APP and BACE1 transcripts following the indicated treatment conditions. Data are mean ± SEM of *n* = 3–6 independent experiments per condition. (c) *APP* mRNA binding to hnRNP C was measured by RT‐qPCR. Data are mean ± SEM of *n* = 4–6 experiments per condition. *P< 0.05, one‐way ANOVA with Tukey post‐hoc comparisons. (d) Representative Western blot showing total hnRNPC and phosphorylated hnRNPC detected using Ab‐Dynabeads to isolate hnRNPC followed by immunoblot using a phosphorylation antibody. Data are mean ± SEM of *n* = 3 experiments per condition. *P < 0.05, one‐way ANOVA with Tukey post‐hoc comparisons. (e) hnRNP C mRNA measured by RT‐qPCR and (f) representative immunoblot and densitometric quantitation of hnRNP C protein levels following the indicated treatments Data are mean ± SEM of *n* = 3‐6 independent experiment per condition. *P < 0.05, **P < 0.01, one‐way ANOVA with Tukey post‐hoc comparisons

We next determined if astrocyte derived EV‐IL‐1β and astrocyte derived EV‐CR modified the expression of APP and the initiating enzyme for the amyloidogenic processing of APP. Protein expression of APP, but not BACE‐1 was increased in neurons exposed to astrocyte derived EV‐IL‐1β compared with astrocyte derived EV‐CR (Figure 2a). We found that the mRNA expression of APP (and BACE) was not increased by astrocyte derived EV‐IL‐1β (Figure 2b), suggesting that a post‐transcriptional regulation of *APP* mRNA was responsible for the observed increase in APP protein expression. APP translation is known to be enhanced by the heterogeneous nuclear ribonuclear protein C (hnRNP C), an RNA binding protein that competitively binds *APP* mRNA, displacing the translational repressor fragile X mental retardation protein (FMRP) to enhance APP translation (Bae et al., 2014; Lee et al., 2010). Astrocyte derived EV‐IL‐1β induced a transient increase in the binding of hnRNP C to *APP* mRNA with a peak at 6 h, and recovery to baseline within 12 h (Figure 2c). This transient binding was accompanied by an increase in hnRNPC mRNA and protein expression with evidence of increased activation by phosphorylation (Figure 2d–f). These data suggest that astrocyte derived EV‐IL‐1β promotes APP translation through increased expression and binding of hnRNP C to APP mRNA.

### 3.3 Astrocyte derived EV‐IL‐1β up‐regulates neuronal APP amyloidogenic processing

**FIGURE 3:**
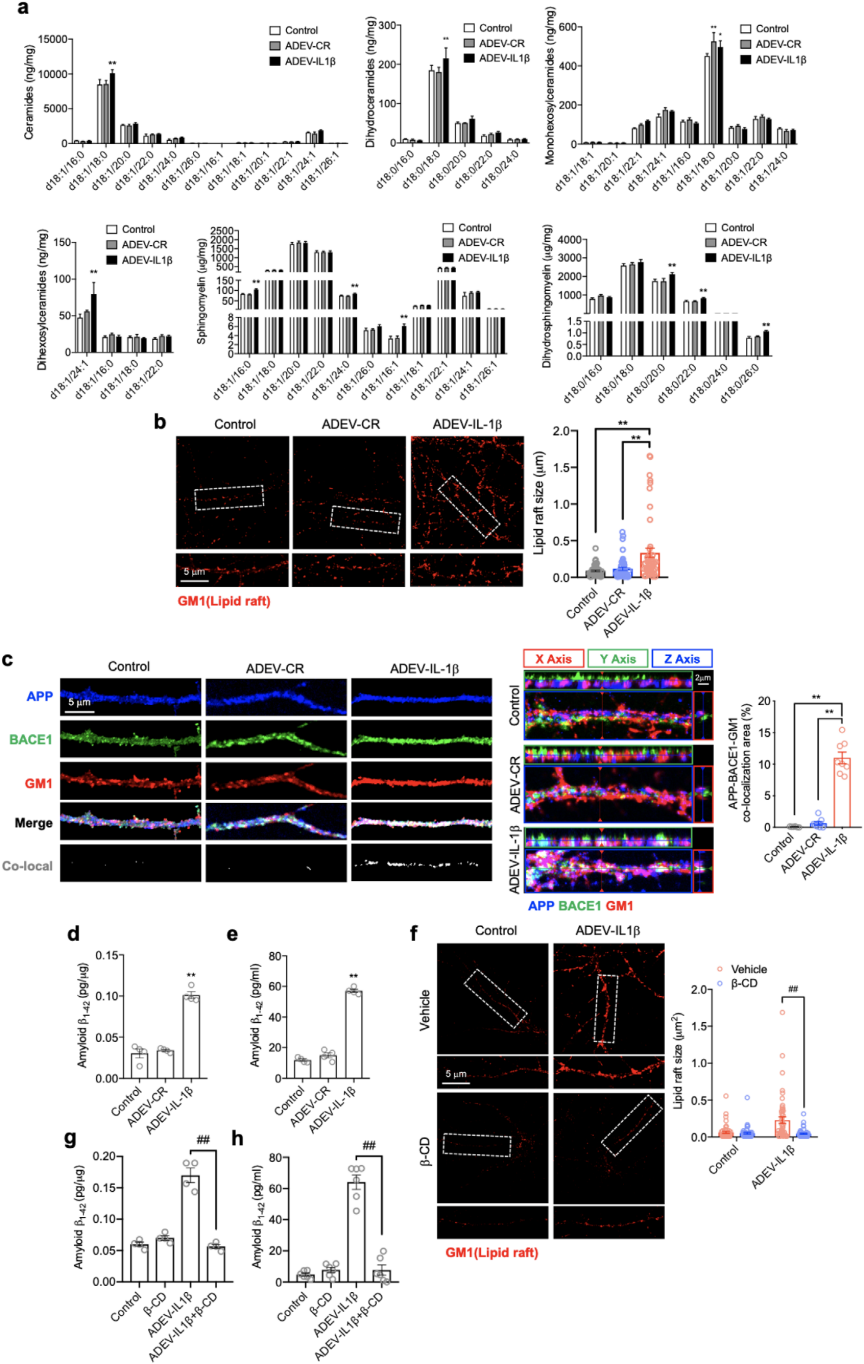
Astrocyte derived EV‐IL‐1β up‐regulated neuronal APP amyloidogenic processing. Cultures were treated with vehicle (control), astrocyte derived EV‐CR (ADEV‐CR) or astrocyte derived EV‐IL‐1β (ADEV‐IL‐1β) for 24 h. (a) Ceramides, dihydroceramides, monohexosylceramides, dihexosylceramides, sphingomyelin and dihydrosphingomyelin were quantitatively measured by mass spectrometry. Data are mean ± SEM of *n* = 3–5 independent experiments per condition. *P < 0.05, **P < 0.01, one‐way ANOVA with Tukey post‐hoc comparisons. (b) Representative images showing cholera toxin submit B staining (labels GM1) in primary neurons and quantitation of lipid raft size following the indicated treatments. Data are mean ± SEM of *n* = 50 dendritic branches in each of three independent experiments. *P < 0.05, **P < 0.01, one‐way ANOVA with Tukey post‐hoc comparisons. (c) Representative immunofluorescent images of primary neurons stained with APP (blue), BACE1 (green), GM1 (red). Merged images show the co‐localization of APP, BACE1 and GM1 as white. Orthogonal views of Z‐stack images show protein co‐localization. Bar graph shows the quantitation of percent co‐localized area along dendritic branches. Data are mean ± SEM of *n* = 8. *P < 0.05, **P < 0.01, one‐way ANOVA. (d‐e) The differentiated SH‐SY5Y (10 μM retinoic acid treated for 7 days) were treated with astrocyte derived EV‐IL‐1β or astrocyte derived EV‐CR for 24 h. The concentration of whole cell lysis (d) and supernatant (e) of astrocyte derived EVs induced differentiated SH‐SY5Y amyloid β_1‐42_ were measured by human Aβ_1‐42_ ELISA. The graphs show mean ± SEM, *n* = 4. **P < 0.01, one‐way ANOVA with Tukey post‐hoc comparisons. (f‐h) Astrocyte derived EV‐IL‐1β companied with β‐cyclodextrin (10 μM) treated neurons for 24 h. The lipid raft size was measured by GM1 immunofluorescence (f), the graphs show mean ± SEM, *n* = 50. *P < 0.05, **P < 0.01, one‐way ANOVA with Tukey post‐hoc comparisons. The concentration of whole cell lysis (g) and supernatant (h) of differentiated SH‐SY5Y human amyloid β_1‐42_ were measured by ELISA. The graphs show mean ± SEM, *n* = 4‐6. ^##^ *P *< 0.01, one‐way ANOVA with Tukey post‐hoc comparisons

A considerable amount of evidence has demonstrated that the α‐cleavage of APP preferentially occurs outside of ceramide‐rich membrane domains, and the amyloidogenic processing of APP occurs primarily in ceramide‐enriched membrane microdomains (Ehehalt et al., 2003; Lee et al., 1998) where the relevant proteins for amyloidogenic processing of APP are concentrated (Kawarabayashi, 2004; Marlow et al., 2003; Parkin et al., 1999; Wahrle et al., 2002; Watanabe et al., 2004). We found that exposing neurons to astrocyte derived EV‐IL‐1β for 24 h increased the ceramide and sphingomyelin content of neurons compared with cells exposed to astrocyte derived EV‐CR (Figure 3a). This increase in ceramides and sphingomyelins were associated with increased size of GM1 immunopositive membrane microdomains domains from 0.089 ± 0.01 μm^2^ in control conditions to 0.3342 ± 0.063 μm^2^ in astrocyte derived EV‐IL‐1β treated neuronal cultures (Figure 3b). We also detected increased co‐localization of APP with BACE‐1 in these enlarged membrane microdomains (Figure 3c), concurrent with increased cytosolic and exported Aβ_1‐42_, and an increase in C‐terminal fragments (Figures 3d–e and S1b). Although rodent cells express the machinery to create Aβ peptides, they are less efficient than human cells in producing Aβ and the Aβ produced does not oligomerize in same manner as human Aβ. For experiments where the production of Aβ is measured we used neuronally differentiated SH‐SY5Y human cells. Disruption of neuronal membrane microdomains with β‐cyclodextrin (β‐CD) after astrocyte derived EV‐IL‐1β exposure reversed the enlargement of membrane microdomains, and reduced the formation of Aβ_1‐42_ (Figure 3f–h). Astrocyte derived EV‐CR did not modify the ceramide content of neurons, membrane microdomain size, or the cellular co‐localization of APP with BACE‐1 (Figure 3a–e). Astrocyte derived EVs shed in response to IL‐1β did not contain IL‐1β protein cargo (Figure S1a), and the direct exposure of neurons to IL‐1β did not modulate the expression of APP, the cellular co‐localization of APP with BACE‐1 in membrane microdomains, or the amount of Aβ_1‐42_ produced (Figure S2a–d). These data demonstrate that IL‐1β is not contained in EVs shed from astrocytes stimulated with IL‐1β, and IL‐1β does not directly promote the amyloidogenic processing of APP in neurons. However, astrocyte derived EV‐IL‐1β promotes the stabilization APP with BACE‐1 in ceramide rich membrane microdomains with a consequent increase in Aβ_1‐42_.

### 3.4 CK1 carried in astrocyte derived EV‐IL‐1β promotes the phosphorylation and binding of hnRNP C to *APP* mRNA

**FIGURE 4:**
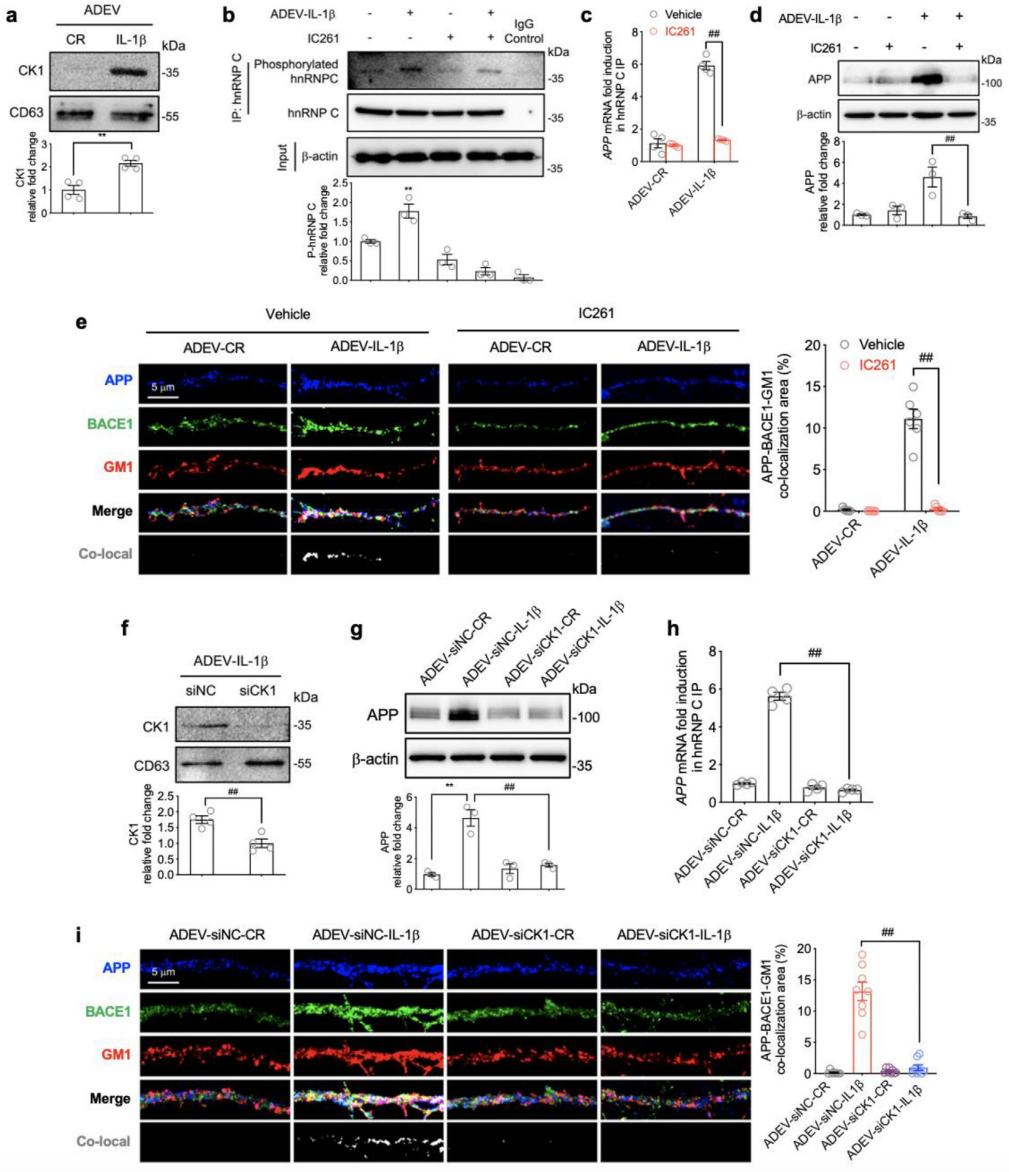
CK1 carried in astrocyte derived EV‐IL‐1β promotes the phosphorylation and binding of hnRNP C to APP mRNA. (a) Representative Western blot and densitometric quantitation of CK1 protein expression in astrocyte derived EV‐CR (ADEV‐CR) and astrocyte derived EV‐ IL‐1β (ADEV‐IL‐1β). Data are mean ± SEM of *n* = 4 independent experiments per condition. **P < 0.01. Student's t‐test. (b) Representative Western blot and densitometric quantitation of total and phosphorylated hnRNP C from neurons treated with vehicle, astrocyte derived EV‐IL‐1β (50 EVs/neuron), the CK1 inhibitor IC261(30 μM), or astrocyte derived EV‐IL‐1β+IC261 for 6 h. Data are mean ± SEM of *n* = 3 independent experiments per condition. **P < 0.01, one‐way ANOVA with Tukey post hoc comparisons. (c) *APP* mRNA binding to hnRNP C measured by RT‐qPCR 6 h following the exposure of neurons to the indicated treatment conditions. Data are mean ± SEM of *n* = 4 independent experiments per condition. **P < 0.01, one‐way ANOVA with Tukey post hoc comparisons. (d) Representative Western blot and densitometric quantitation of APP from neurons treated with vehicle, astrocyte derived EV‐IL‐1β (50 EVs/neuron), the CK1 inhibitor IC261 (30 μM), or astrocyte derived EV‐IL‐1β+IC261 for 24 h. Data are mean ± SEM of *n* = 3 independent experiments per condition. **P < 0.01, one‐way ANOVA with Tukey post hoc comparisons. (e) Immunofluorescent images of dendritic branches showing APP (blue), BACE1 (green), GM1 (red) and the merged images. Co‐localized APP, BACE1 and GM1 are shown in white. Bar graph shows the quantitation of percent co‐localized area along dendritic branches. Data are mean ± SEM of *n* = 6 independent experiments per condition. ^##^ *P *< 0.01, one‐way ANOVA with Tukey post‐hoc comparisons. (f) Representative Western blot and densitometric analysis of astrocyte derived EV‐IL‐1β isolated from astrocytes transfected with a small interfering non‐coding RNA (siNC) or siCK1. Data are mean ± SEM of *n* = 4 independent experiments per condition. ^##^ *P *< 0.01. Student's t‐test. (g) Representative Western blot and densitometric quantitation of APP from neurons treated with astrocyte derived EV‐siNC‐CR (ADEV‐siNC‐CR), astrocyte derived EV‐siNC‐IL1β (ADEV‐siNC‐IL1β), astrocyte derived EV‐siCK1‐CR (ADEV‐siCK1‐CR), or astrocyte derived EV‐siCK1‐IL1β (ADEV‐siCK1‐IL1β). Data are mean ± SEM, *n* = 3 independent experiments per condition. **P < 0.01, ^##^ *P *< 0.01. One‐way ANOVA with Tukey post hoc comparisons. (h) *APP* mRNA binding to hnRNP C measured by RT‐qPCR 6 h following the exposure of neurons to the indicated treatment conditions. Data are mean ± SEM of *n* = 4 independent experiments per condition. (i) Immunofluorescent images of dendritic branches 24 h following the indicated treatment conditions showing APP (blue), BACE1 (green), GM1 (red) and the merged images. Co‐localized APP, BACE1 and GM1 are shown in white. Bar graph shows the quantitation of percent co‐localized area along dendritic branches. Data are mean ± SEM of *n* = 8 independent experiments per condition. ^##^ *P *< 0.01. One‐way ANOVA with Tukey post hoc comparisons

We next sought to determine the mechanism by which astrocyte derived EV‐IL‐1β promotes the activation (phosphorylation) of hnRNP C. We searched a previously conducted proteomic analysis of astrocyte derived EV‐IL‐1β (Datta Chaudhuri et al., 2020) to identify potential hnRNP C interacting proteins and identified casein kinase 1 (CK1) as a potential mediator of hnRNP C phosphorylation (Kattapuram et al., 2005). We found that CK1 was packaged into astrocyte derived EV‐IL‐1β, but was not detected in astrocyte derived EV‐CR (Figure 4a). Pre‐treatment of neuronal cultures with the CK1 inhibitor IC261 (Mashhoon et al., 2000) prevented astrocyte derived EV‐IL‐1β from increasing the phosphorylation and binding of hnRNP C to *APP* mRNA (Figures 4b and 4c), blocked the increase in APP protein expression (Figure 4d), and prevented the increased co‐localization of APP with BACE1 in membrane microdomains (Figure 4e). We confirmed that these observations were the result of CK1 delivery to neurons using siRNA to specifically reduce CK1 expression in astrocytes (Figure S3a), and then stimulated the release of astrocyte derived EVs from these cells with IL‐1β (astrocyte derived EV‐siCK1‐IL‐1β). CK1 was not present in astrocyte derived EV‐siCK1‐IL‐1β (Figure 4f). CK1‐deficient astrocyte derived EVs did not increase the binding of hnRNP C to *APP* mRNA, and did not increase APP expression or the co‐localization of APP with BACE1 in membrane microdomains (Figure 4g–i). Astrocyte derived EV‐IL‐1β from astrocytes pre‐exposed to a scrambled siRNA (astrocyte derived EV‐siNC‐IL‐1β) produced results in discriminable from astrocyte derived EV‐IL‐1β that included increased binding of hnRNP C to *APP* mRNA, increased APP expression, and increased co‐localization of APP with BACE1 in membrane microdomains (Figure 4g–i). These data demonstrate that IL‐1β stimulation of astrocytes promotes the packaging of CK1 into astrocyte derived EVs that are delivered to neurons where they promote increased APP expression.

### 3.5 Confirmation that CK1 carried in astrocyte derived EV‐IL‐1β is delivered into target neurons

**FIGURE 5:**
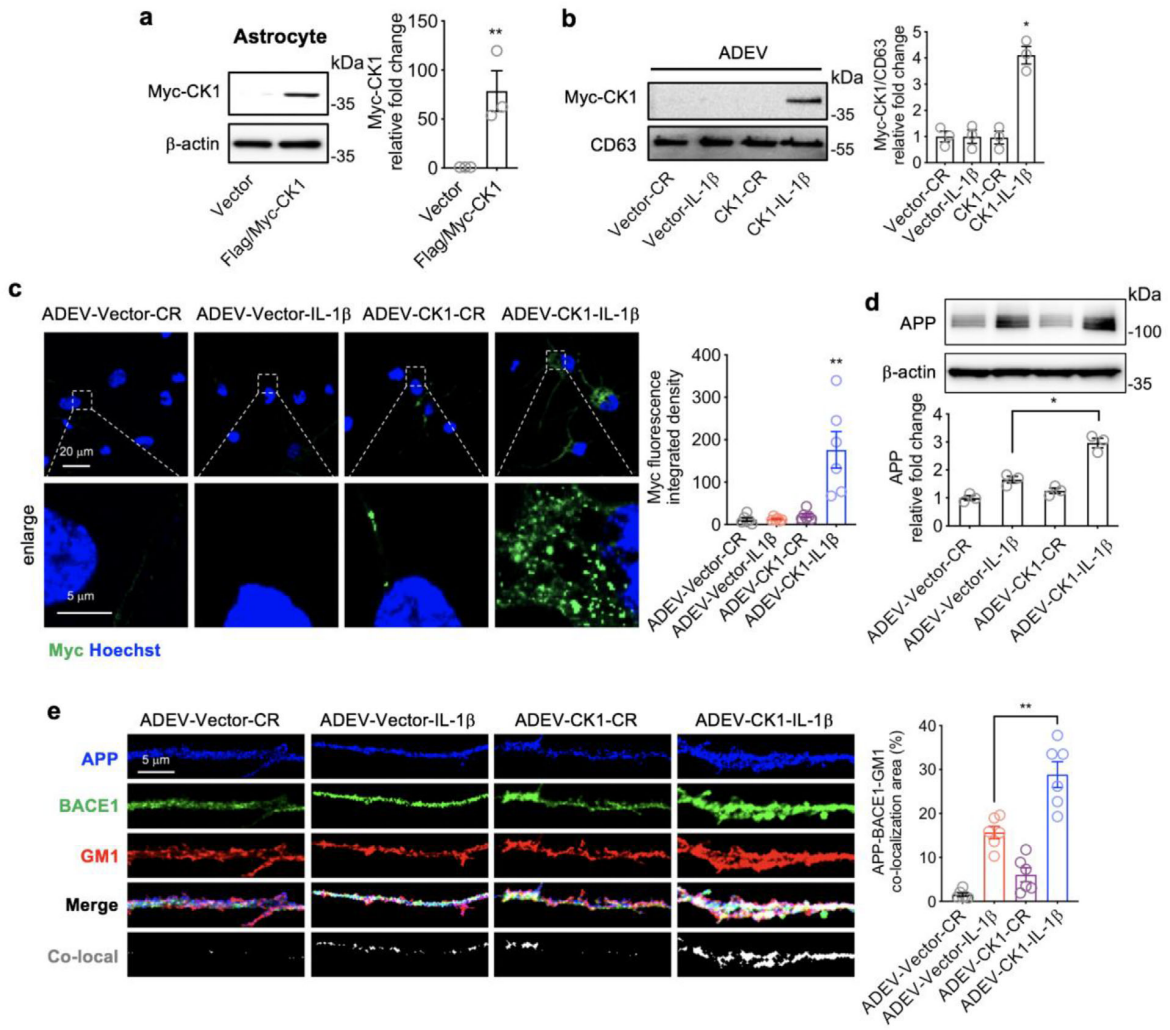
Confirmation that CK1 carried in astrocyte derived EV‐IL‐1β is delivered into target neurons. (a) Representative Western blot and quantitative densitometry of astrocytes transfected with an empty vector or a vector expressing Myc tagged CK1. Data are mean ± SEM of *n* = 3 independent experiments per condition. **P < 0.01, one‐way ANOVA with Tukey pos‐hoc comparison. (b) Representative western blot and quantitative densitometry of EVs isolated from the media of astrocytes transfected with and empty vector or a vector expressing Myc‐Tagged‐CK‐1. EVs were constitutively released (CR), or shed in response to IL‐1β as indicated. Data are mean ± SEM of *n* = 3 independent experiments per condition. *P < 0.05, one‐way ANOVA with Tukey post hoc comparisons. (c) Representative immunofluorescent images and quantitation of fluorescent intensity of neurons treated with astrocyte derived EV‐Vector‐CR (ADEV‐Vector‐CR), astrocyte derived EV‐Vector‐IL1β (ADEV‐Vector‐IL1β), astrocyte derived EV‐CK1‐CR (ADEV‐CK1‐CR), and astrocyte derived EV‐CK1‐IL1β (ADEV‐CK1‐IL1β) for 3 h then stained for anti‐Myc. Data are mean ± SEM of *n* = 6 independent experiments per condition. **P < 0.01, one‐way ANOVA with Tukey post hoc comparisons. (d‐e) Neurons were treated with astrocyte derived EV‐Vector‐CR, astrocyte derived EV‐Vector‐IL1β, astrocyte derived EV‐CK1‐CR, and astrocyte derived EV‐CK1‐IL1β for 24 h and APP expression was measured by (d) Western blot and (e) immunofluorescence. Representative immunofluorescent images of dendritic branches following the indicated treatment conditions showing APP (blue), BACE1 (green), GM1 (red) and the merged images. Co‐localized APP, BACE1 and GM1 are shown in white. Data are mean ± SEM of *n* = 3 (Western blot) and *n* = 6 (fluorescence). *P < 0.05, one‐way ANOVA with Tukey post hoc comparisons

We next sought to validate that the CK1 cargo of astrocyte derived EV‐IL‐1β was responsible for the increased expression of hnRNP C. We transfected astrocytes with a Myc‐tagged CK1 plasmid, or an empty vector plasmid (pCMV6) and confirmed 48 h post‐transfection that astrocytes express Myc‐tagged CK1 by immunoblotting (Figure 5a) and immunofluorescence staining (Figure S3b). Stimulation of transfected cells with IL‐1β produced astrocyte derived EVs with Myc‐tagged CK1 (astrocyte derived EV‐CK1‐IL‐1β), further confirming that IL‐1β simulation of astrocytes promotes the inclusion of CK1 into astrocyte derived EVs. As expected, Myc‐tagged CK1 was not present in EVs constitutively shed from astrocytes transfected with Myc‐tagged CK1 (astrocyte derived EV‐CK‐CR) (Figure 5b). Astrocytes transfected with an empty vector were either treated with IL‐1β (astrocyte derived EV‐Vector‐IL‐1β), or allowed to constitutively shed EVs (astrocyte derived EV‐Vector‐CR) as controls. In neurons exposed to astrocyte derived EV‐CK1‐IL‐1β, we observed immunopositive staining for Myc‐tagged CK1 in neuronal cell bodies and dendrites (Figure 5c). There was no visible uptake of Myc‐tagged CK1 in neurons exposed to astrocyte derived EV‐CK1‐CR, or when empty vector was delivered with astrocyte derived EV‐Vector‐CR, or astrocyte derived EV‐Vector‐IL‐1β (Figure 5c). The expression of APP, and the co‐localization of APP with BACE1 in membrane microdomains was increased by astrocyte derived EV‐CK1‐IL‐1β (Figures 5d and 5e) suggesting that the Myc tag did not modify the function of CK1.

### 3.6 CK1 carried in astrocyte derived EV‐IL‐1β directly binds GSK3α/β and APC in target neurons

**FIGURE 6:**
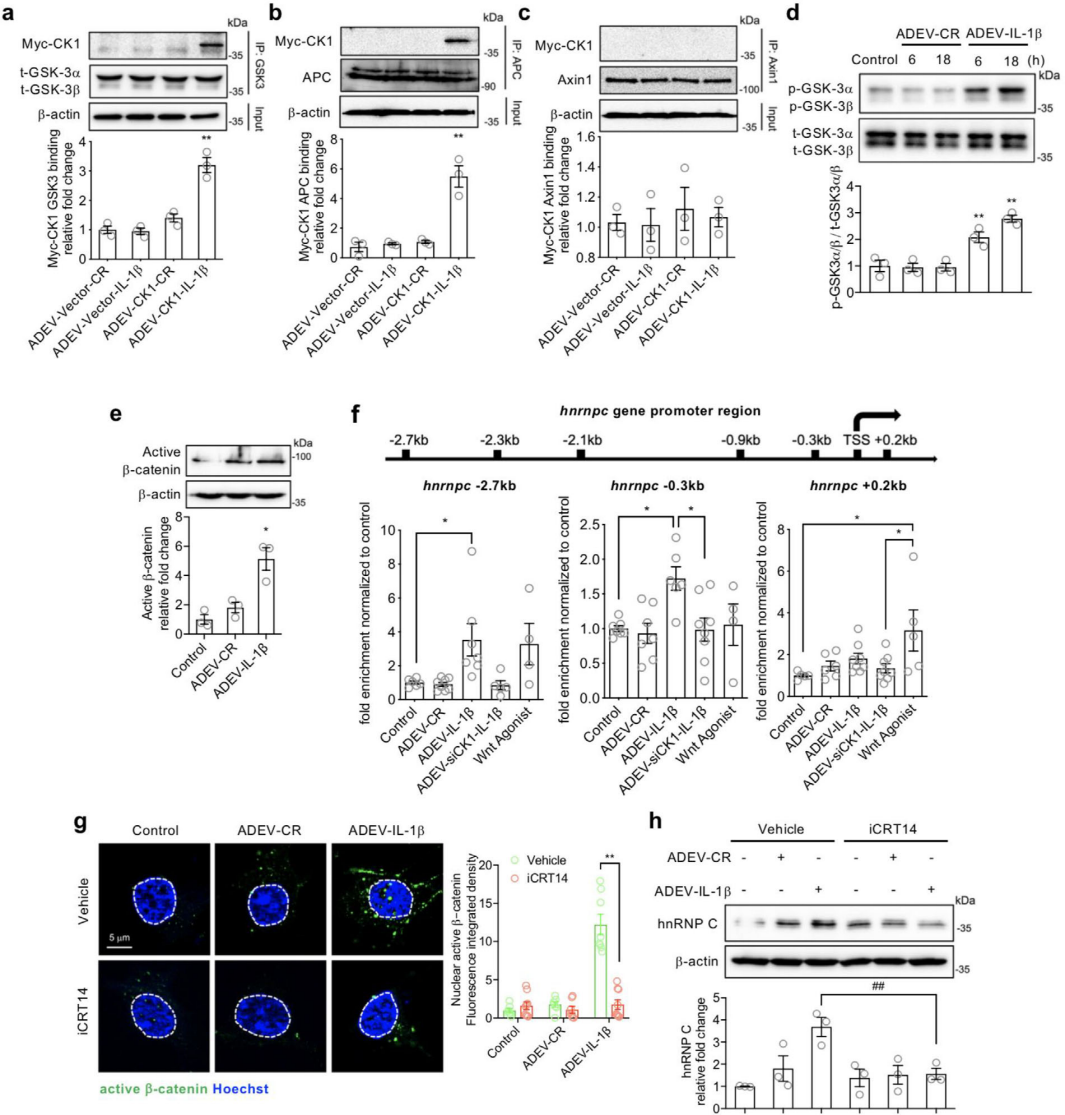
CK1 carried in astrocyte derived EV‐IL1β directly binds to GSK3α/β and APC in target neurons. Neurons were treated with astrocyte derived EV‐Vector‐CR (ADEV‐Vector‐CR), astrocyte derived EV‐Vector‐IL1β (ADEV‐Vector‐IL1β), astrocyte derived EV‐CK1‐CR (ADEV‐CK1‐CR), and astrocyte derived EV‐CK1‐IL1β (ADEV‐CK1‐IL1β) for 18 h. (a) GSK3‐Myc‐CK1, (b) Axin1‐Myc‐CK1 and (c) APC‐Myc‐CK1 were immunoprecipitated and the immunoblot probed for Myc tagged CK1. Myc‐CK1 was quantified by band density. (d) Neurons were treated with astrocyte derived EV‐CR (ADEV‐CR) or astrocyte derived EV‐IL1β (ADEV‐IL1β) for 6 and 18 h, Total GSK‐3α,β and ‐GSK3α,β were detected by Western blot and quantified by densitometry. (e) Neurons were treated with astrocyte derived EV‐CR or astrocyte derived EV‐IL‐1β for 18 h and active β‐catenin expression was measured by Western blot and quantified by densitometry. (f) Neurons were treated with astrocyte derived EV‐CR (ADEV‐CR), astrocyte derived EV‐IL1β (ADEV‐IL‐1β), astrocyte derived EV‐siCK1‐IL1β (ADEV‐siCK1‐IL‐1β), and Wnt agonist (20 μM) for 18 h. The binding of β‐catenin to *Hnrnpc* promotor sites at ‐2.7, ‐0.3 and +0.2 kb were measured by chromatin immunoprecipitation ChIP‐qPCR analysis. Data are mean ± SEM of *n* = 3‐8 independent experiments per condition. *P < 0.05, **P < 0.01, one‐way ANOVA with Tukey post hoc comparisons. (g) Neurons were treated with astrocyte derived EV‐CR, astrocyte derived EV‐IL1β in the presence and absence of the β‐catenin inhibitor iCRT (20 μM) for 18 h. The nuclear translocation of β‐catenin was visualized by immunofluorescence and quantified by fluorescence intensity. Nuclear staining with Hoescht33342 is shown as blue and active β‐catenin is green. Data are mean ± SEM of *n* = 8 independent experiments per condition. **P < 0.01, one‐way ANOVA with Tukey post hoc comparisons. (h) Neuronal hnRNP C expression was detected by Western blot and quantified by densitometry following the indicated treatment conditions. Data are mean ± SEM of *n* = 3 independent experiments per condition **P < 0.01, one‐way ANOVA with Tukey post hoc comparisons

CK1 is part of the β‐catenin degradation complex that participates in Wnt/β‐catenin signalling (Cruciat, 2014; Li et al., 2012). In the absence of Wnt stimulation, Axin1, APC, GSK3 and CK1 form a complex with β‐catenin that facilitates the degradation of β‐catenin. With Wnt stimulation this degradation complex is inhibited, β‐catenin is stabilized and translocated to the nucleus where it acts as a transcriptional promoter (Clevers & Nusse, 2012; Niehrs, 2012). We next determined if CK1 delivered in astrocyte derived EVs formed a complex with APC, Axin1 and GSK3α/β in neurons. Neurons were exposed to astrocyte derived EV‐Vector‐CR, astrocyte derived EV‐Vector‐IL‐1β, astrocyte derived EV‐CK1‐CR, or astrocyte derived EV‐CK1‐IL‐1β and imaged using a fluorescently conjugated antibody directed against Myc in combination with fluorescently tagged antibodies directed against APC, Axin1 and the serine‐threonine kinase GSK3α/β. In neurons treated with astrocyte derived EV‐CK1‐IL‐1β Myc‐tagged CK1 co‐localized with GSK3α/β, and APC, but not Axin1 in neuronal cell bodies and dendrites suggesting they are at least closely associated with one another (Figure S4a–c). Co‐immunoprecipitation experiments confirmed that CK1 delivered to neurons in astrocyte derived EV‐IL‐1β directly interacts with neuronal GSK3α/β, and APC, but not Axin1 (Figure 6a–c). Compared to astrocyte derived EV‐CR, the treatment of neurons with astrocyte derived EV‐IL‐1β produced a time‐dependent increase in GSK‐3 phosphorylation on serine 21 and GSK‐3β on serine 9 (Figure 6d), and an increase in unphosphorylated (active) β‐catenin (Figures 6e and S4d). These findings are consistent with a CK1‐mediated de‐stabilization and inactivation of the Wnt signalling degradation complex. Indeed, astrocyte derived EV‐IL‐1β, but not astrocyte derived EV‐CR promoted the translocalization of β‐catenin from the cytosol to the nucleus in neurons (Figure 6g) where it bound the promoter region of hnRNP C.

To identify the binding sites of β‐catenin at the *Hnrnpc* gene we scanned the sequences from ‐3 kb to +0.5 kb from the transcription start site (TSS) of the *Hnrnpc* gene. The predicted binding sites based on TCF/LEF consensus sequences were ‐2.7, ‐2.3, ‐2.1, ‐0.9, ‐0.3 kb, TSS, and +0.2 kb. A β‐catenin ChIP‐qPCR demonstrated that astrocyte derived EV‐IL‐1β induced the binding of β‐catenin at ‐2.7 and ‐0.3 kb from *Hnrnpc* gene transcription start sites (Figures 6f and S4e). In contrast, stimulating neurons with a Wnt agonist promoted β‐catenin binding at +0.2 kb from the *Hnrnpc* gene transcription start sites (Figure 6f). β‐Catenin binding to any sites of the *Hnrnpc* promotor region was not observed when neurons were treated with astrocyte derived EV‐siCK1‐IL‐1β, confirming that CK1 delivered in astrocyte derived EV‐IL‐1β was responsible for promoting binding of β‐catenin at the ‐2.7 and ‐0.3 kb *Hnrnpc* gene transcription start sites (Figures 6f and S4e). The nuclear translocation of β‐catenin and expression of hnRNP C was blocked by pre‐treatment of neurons with iCRT14, a potent inhibitor of β‐catenin‐responsive transcription (Figures 6g and 6h). These data demonstrate that CK1 carried in astrocyte derived EV‐IL‐1β directly binds to GSK3α/β and APC in target neurons promoting the stabilization and nuclear translocation of β‐catenin that binds to *Hnrnpc* gene promoter regions. This effect produced by the direct delivery of CK1 to neurons in astrocyte derived EVs is distinct from β‐catenin binding induced by known canonical and non‐canonical Wnt signalling that does not target *Hnrnpc* gene promoter regions.

### 3.7 CK1 enriched on astrocyte‐derived extracellular vesicles in Alzheimer's disease

**FIGURE 7:**
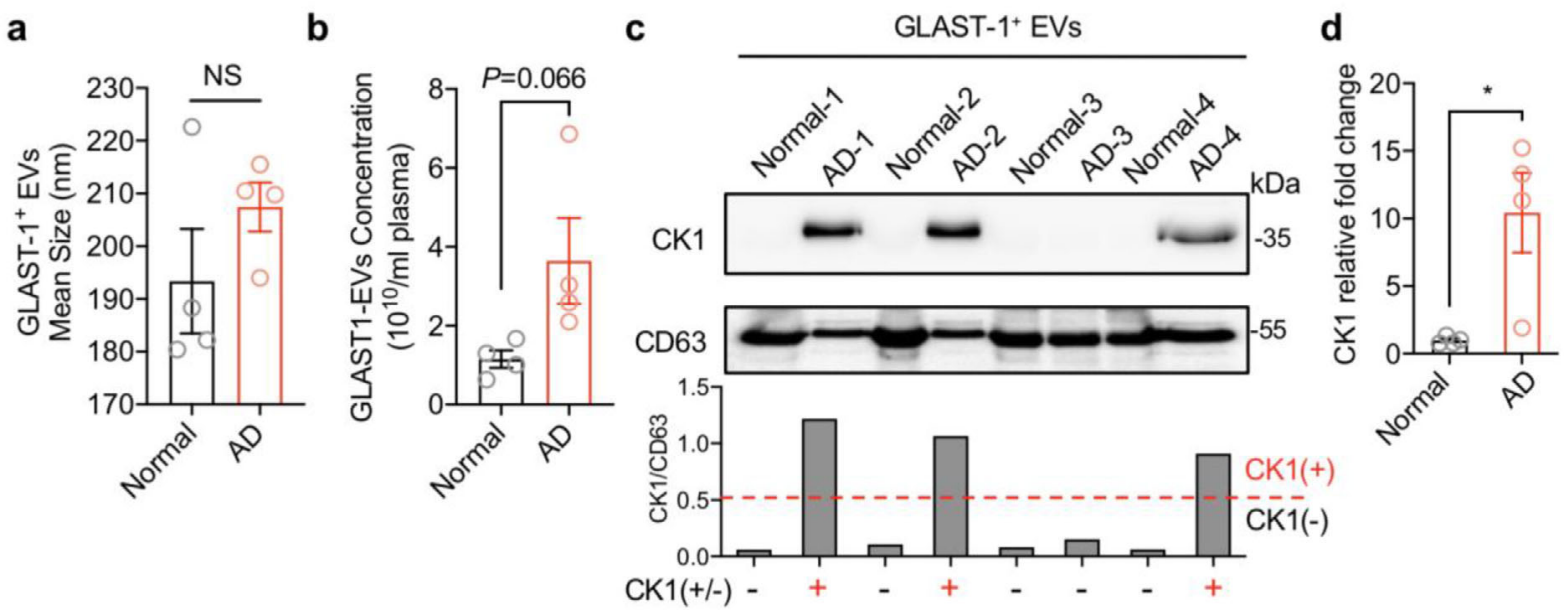
CK1 enriched on astrocyte‐derived extracellular vesicles in Alzheimer's disease. GLAST‐1^+^ EVs were isolated from patients with Alzheimer's disease and age‐matched health control subjects. (a) EV size and (b) concentration were calculated by nanoparticles tracking analysis. NS = no significant difference. (c) CK1 and CD63 were detected by Western blot and (d) quantified by densitometry. A CK1/CD63 ratio >0.5 was defined as CK1 positive and quantified as relative fold change Data are mean ± SEM of *n* = 4 independent experiments per condition. *P < 0.05, Student's t‐test

We isolated GLAST‐1 immunopositive EVs from the plasma of 4 patients with CSF biomarker evidence for Alzheimer's disease and four sex‐ and age‐matched cognitively normal control subjects. The mean size (Figures 7a and S5a) and concentration (Figures 7b and S5a) of GLAST‐1 immunopositve EVs were not significantly different in the two groups. However, CK1 was detected in GLAST‐1 immunopositive EVs from AD patients (three out of four) compared to control subjects (zero out of four) (Figures 7c and 7d).

## 4 DISCUSSION

There is a considerable amount of evidence that the deposition of Aβ peptides in brain parenchyma begins during the earliest stages of AD. However, most of what we know about inflammation in AD stems from the pro‐inflammatory effects of pathological proteins. For example, there is a considerable amount of evidence that Aβ_1‐42_ and other amyloidogenic peptides promote a pro‐inflammatory phenotype in brain (Abbas et al., 2002; Fiala et al., 1998; Tehranian et al., 2001) that is largely attributed to the activation of TLRs and RAGE receptors on microglial cells and astrocytes (Arancio et al., 2004; Reed‐Geaghan et al., 2009). Ligation of these pattern recognition receptors results in activation of the transcription factors NF‐κB and AP‐1 that up‐regulate the expression of numerous inflammatory cytokines, including IL‐1β, IL‐6 and TNF‐α (Benzing et al., 1999; Bonaiuto et al., 1997; Vukic et al., 2009). These pro‐inflammatory mediators have direct effects on astrocytes and adjacent microglia to promote an A1 phenotype and amplify pro‐inflammatory signals (Liddelow et al., 2017; Meraz‐Ríos et al., 2013). Although IL‐1β has been shown to increase APP expression in endothelial cells (Goldgaber et al., 1989), and IL‐6 increases APP expression in mixed neuronal‐glial cultures (Del Bo et al., 1995), there is no evidence that inflammatory cytokines directly promote the amyloidogenic processing of APP in neurons with the exception of one study that found the combination of TNFα and IFNγ triggers the production of Aβ peptides and inhibits the secretion of soluble APPs in Sk‐n‐sh neuronal cells and thyroid epithelial cells (Blasko et al., 1999). Similar to the vast majority of reported findings, we found that IL‐1β, TNFα and IFNγ did not stimulate amyloidogenic processing of APP when directly applied onto neuronal cultures. However, we did find that astrocyte derived EVs shed in response to IL‐1β increased the production of Aβ_1‐42_ when directly applied onto neuronal cultures. Our data suggest that chronic inflammation may promote the amyloidogenic processing of APP through an indirect mechanism that involves the packaging of CK1 into astrocyte derived EVs that promote ligand‐independent Wnt signalling in neurons.

**FIGURE 8:**
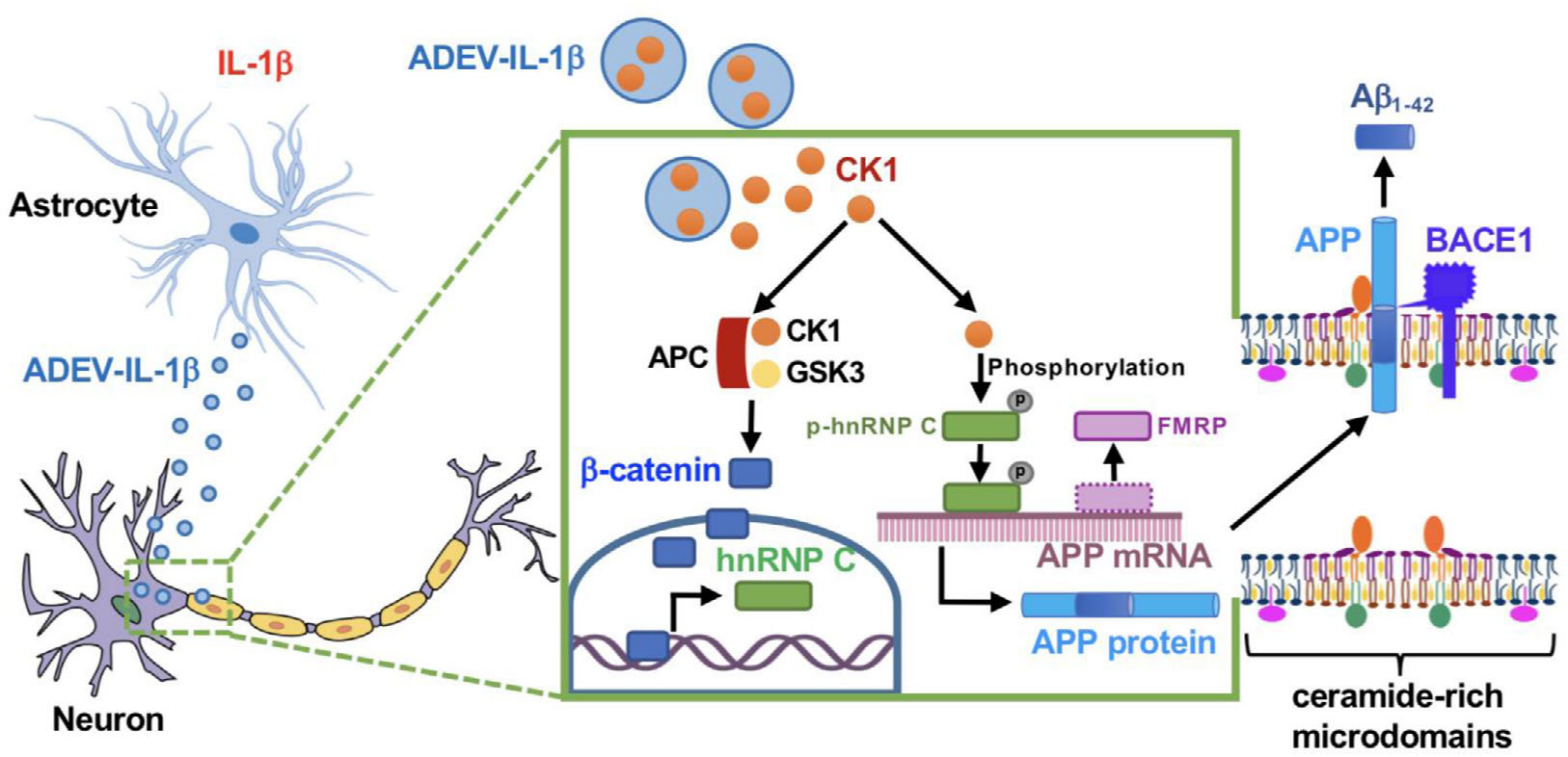
Schematic model of astrocyte derived EV‐IL‐1β promoted neuronal APP amyloidogenic processing. Astrocytes stimulated with IL‐1β (astrocyte derived EV‐IL‐1β, ADEV‐IL‐1β) shed extracellular vesicles that are enriched with CK1. Astrocyte derived EV‐IL‐1β is delivered to neurons where the CK1 originating from astrocytes binds with neuronal APC and GSK to inhibit the β‐catenin degradation complex. Stabilized β‐catenin translocates to the nucleus and binds the *Hnrnpc* gene promoter regions ‐2.7 and ‐0.3 kb to enhance hnRNP C expression. This binding is fundamentally different from β‐catenin binding induced by classical Wnt signaling. The hnRNP C protein dislocates the translational repressor FMRP and binds to APP mRNA promoting the translation of *APP* mRNA. APP protein accumulates in membrane microdomains with BACE1 to promote the amyloidogenic processing of APP into Aβ peptides

Roles for EVs in the pathogenesis of AD are emerging, but are controversial. EVs have been reported to carry Aβ, APP, APP‐CTFs (C‐terminal fragments) and AICD (APP intercellular domain) (Perez‐Gonzalez et al., 2012; Sharples et al., 2008; Vingtdeux et al., 2007). However, it is not clear if EVs play a role in extracellular deposition, or the clearance of Aβ peptides. Recent findings from animal model systems suggest that EVs may be responsible for the prion‐like spread neurotoxic proteins including Tau and Aβ (Baker et al., 2016; Crotti et al., 2019; Dinkins et al., 2016; Rajendran et al., 2006; Sardar Sinha et al., 2018), and lipids contained in EVs shed from microglia may promote the extracellular formation of neurotoxic soluble Aβ species from extracellular insoluble aggregates (Joshi et al., 2014). Attenuation of whole body EVs release through pharmacological inhibition or genetic mutation of the sphingomyelin phosphodiesterase neutral sphingomyelinase‐2 (nSMase2) in the 5XFAD mouse reduces Aβ plaque load and improves performance in a behavioural fear conditionings task (Dinkins et al., 2014, 2016). Although these data suggest that EVs may play a detrimental role in the deposition of Aβ, other studies have demonstrated that EVs are rich in glycosphingolipids that bind Aβ and promote clearance by delivering these amyloidogenic peptides to microglia for phagocytotic degradation (Yuyama et al., 2014, 2015). Likewise, N2a and SH‐SY5Y cells derived exosomes are enriched with cellular prion protein that is known to bind oligomeric Aβ_42_ and promote fibrillization (Falker et al., 2016). Indeed, EVs readily cross the blood brain barrier and enter into peripheral circulation (Dickens et al., 2017). These models are further complicated when one also considers interactions of Aβ peptides with EV biogenesis. For example, Aβ is known to induce nSMase2 activity (Lee et al., 2004), and this enzyme is important for the biogenesis of EVs (Trajkovic et al., 2008). EVs shed in response to Aβ stimulation of neurons are enriched with PAR‐4 and ceramide that promote apoptosis in astrocytes (Wang et al., 2012). Despite this growing wealth of evidence on the relationship of EVs to amyloid pathology, EVs have not yet been implicated in the production of Aβ peptides. Here we provide new evidence of a unique signalling mechanism whereby IL‐1β promotes the release of EVs from astrocytes enriched with CK1 that when delivered to neurons increases the expression of APP through a direct interaction of CK1 with the Wnt degradation complex that ultimately stimulates the production and amyloidogenic processing of APP (Figure 8).

One of the most intriguing parts of this study was the discovery that IL‐1β stimulation of astrocytes promotes the packaging of CK1 into astrocyte derived EVs. In support of this findings, CK1 was present in plasma astrocyte derived EVs of most AD patients examined but absent in the control subjects. This EV facilitated delivery of CK1 to neurons was critical for the increased expression of APP and the production of Aβ. We found that CK1 originating from astrocytes formed a complex with neuronal GSK3α/β and APC to de‐stabilize the β‐catenin degradation complex. Although this Wnt‐independent destabilization of the degradation complex promoted the nuclear translocation of β‐catenin, the resulting transcriptional regulation was distinct from known canonical and non‐canonical Wnt signalling. Canonical Wnt signalling has been consistently associated with a reduction in the formation of Aβ, while non‐canonical Wnt signalling (which bypasses the degradation complex) is associated with the increased formation of Aβ (Elliott et al., 2018; Tapia‐Rojas et al., 2016). In our studies we found that the nuclear translocation of β‐catenin was associated with the increased production of Aβ. The mechanism for this unusual property of β‐catenin resulted from forming complexes with TCF/LEF and binding to hnRNP C promotor regions at sites located ‐2.7 and ‐0.3 kb from the transcription start site. This binding was distinct from the promoter regions β‐catenin interacted with following a classical activation of Wnt signalling that promoted β‐catenin binding outside of the hnRNP C gene. The increased transcription of hnRNP C stabilized APP translation. The RNA‐binding proteins hnRNP C and FMRP compete for the same coding region element in *APP* mRNA. APP translation is enhanced when hnRNP C displaces the translational repressor FMRP from binding to *APP* mRNA (Lee et al., 2010). Although this mechanism explains the increase of APP expression in response to astrocyte derived EV‐IL‐1β, it does not explain the increased processing of APP to Aβ.

The enhanced amyloidogenic processing of APP in response to astrocyte derived EV‐IL‐1β occurred through a stabilization of APP with BACE‐1 in ceramide rich membrane microdomains. Amyloid‐β peptides are produced by a sequential proteolytic processing of APP. APP contains a large receptor‐like N‐terminal region that is cleaved by α‐secretase at position Lys687 to generate an 83‐residue peptide, or by β‐secretase at position Asp672 to generate a 99‐residue peptide. Both the 83‐ and 99‐residue peptides are substrates for a third membrane‐associated protease called γ‐secretase (a protein complex of presenilin, nicastrin, APH‐1 and Pen‐2). The proteolysis by γ‐secretase is heterogeneous and generates several Aβ peptides with different length. Although the most abundant species is a 40‐residue peptide (Aβ_1‐40_), a 42‐residue peptide (Aβ_1‐42_) is also formed which is more prone to fibril formation. The cleavage of APP by BACE‐1 preferentially occurs in ceramide and cholesterol‐rich membrane microdomains where BACE‐1 is preferentially located. Cleavage of APP by α‐secretase occurs outside of membrane microdomains where ADAM10 and other α‐secretases are preferentially located (Bodovitz & Klein, 1996; Kojro et al., 2001). Exposure of neurons to astrocyte derived EV‐IL‐1β increased the size of membrane microdomains and stabilized the localization of APP with BACE1. Previous studies have shown that artificially stabilizing membrane microdomains with the addition of cholesterol increases BACE‐1 activity which leads to the increased formation of Aβ (Raffaï & Weisgraber, 2003). Here we found that disrupting the structure of membrane microdomains by removing cholesterol with β‐cyclodextrin reduced the localization of APP with BACE1, and the formation of Aβ (Simons et al., 1998).

The findings from these experiments identify a mechanism where chronic inflammation may indirectly promote the production of Aβ in neurons through the enhanced release of astrocyte derived EVs enriched with CK1. From a mechanistic standpoint, these studies demonstrate that protein cargo of astrocyte derived EVs can be delivered to neurons and functionally interact with neuronal proteins. Astrocyte derived CK1 destabilizes the β‐catenin degradation complex by directly interacting with neuronal GSK3 and APC. This Wnt‐independent signalling results in the nuclear translocation of β‐catenin which interacts with promoter regions distinct from those resulting from the direct activation of classical Wnt signalling. This study reveals an indirect mechanism where inflammatory stimuli promote the release of astrocyte derived EVs that when delivered to neurons promote the amyloidogenic processing of APP. These findings suggest interventions which reduce the release of EVs in the setting of chronic inflammation may delay the onset or slow the progression of AD by reducing the formation of Aβ peptides.

## CONFLICT OF INTEREST

The authors declare that they have no conflict of interest.

## Supporting information



Table S1. Primers used for RT‐qPCR
This table showing the primers sequences used in this study
**Table S2. Primers used for ChIP‐qPCR**
This table showing the primers sequences used for ChIP experiment.
**Table S3. Antibody used in this study**
**WB: Western Blot; IF: Immunofluorescence; IP: immunoprecipitation; ChIP: Chromatin immunoprecipitation**
Table graphs showing the information of Alzheimer's Disease patients’ age, gender, clinical stage, and concentration of CSF Aβ, tau, p‐tau as well as the age and gender matched normal individuals.
**Figure S1 (relate to Figure 2) Astrocyte derived EV‐IL‐1β induced neuronal APP C terminal fragmentation. (a)** Astrocyte derived extracellular vesicles shed in response to IL‐1β (200 ng/ml; astrocyte derived EV‐IL1β as ADEV‐IL‐1β) or CR (astrocyte derived EV‐CR as ADEV‐CR) were isolated from media using a multi‐step ultracentrifugation. Representative Western Blot of IL‐1β is shown for the indicated treatment conditions. Recombinant IL‐1β (0.2μg) was included as a positive control. Data are mean ± SEM of *n* = 3 independent experiments per condition. NS = no significant changes, One‐way ANOVA with Tukey post‐hoc comparisons. **(b)** Neurons were treated with astrocyte derived EV‐CR (ADEV‐CR) and astrocyte derived EV‐IL‐1β (ADEV‐IL‐1β) for 24h. Full‐length (FL‐APP) and C‐Terminal Fragments (APP‐CTFs) of APP were detected by Western Blot using APP (Y188) antibody. Data are mean ± SEM of *n* = 3 independent experiments per condition. * = p < 0.01, One‐way ANOVA with Tukey post‐hoc comparisons.
**Figure S2 (relate to Figure 3). IL‐1β did not modulate APP and Aβ_1‐42_ expression. (a)** 200 ng/ml recombinant IL‐1β treated primary neurons for 24h. The APP protein expression were detected by western blot. **(b)** representative immunofluorescent images of primary neurons showing APP (blue), BACE1 (green), GM1 (red). Merged images show the co‐localization of APP, BACE1 and GM1 as white. Bar graph shows the quantitation of percent co‐localized area along dendritic branches. Data are mean ± SEM of *n* = 6. NS = no significant changes, One‐way ANOVA with Tukey post‐hoc comparisons. **(c‐d)** 200 ng/ml IL‐1β treated differentiated SHSY5Y cells for 24h, the whole cell lysate **(b)** and supernatant **(c)** of Aβ_1‐42_ were measured by human Aβ_1‐42_ ELISA. The graphs show mean ± SEM, *n* = 4‐5. NS = no significant changes. Student's t‐test.
**Figure S3 (relate to Figure 4 and Figure 5). Knockdown or overexpression of CK1 in astrocytes. (a)** 15 or 30 nM of siNC and siCK1 transfected into astrocytes for 48h, the CK1 expression on astrocytes were measured by western blot. The graph bars show mean ± SEM, *n* = 3. ^## ^= *p *< 0.01. One‐way ANOVA with Tukey post hoc comparisons. **(b)** Astrocytes transfected with empty liposome or Myc‐tagged CK1 plasmid for 48 h, the expression of Myc‐CK1 were stained by immunofluorescences using Myc‐tagged antibody. The graphs show mean ± SEM, *n* = 6. ** = *p* < 0.01, Student's t‐test.
**Figure S4 (relate to Figure 6). CK1 carried in astrocyte derived EV‐IL1β directly binds GSK3a/b and APC in target neurons. (a‐d)** Neurons were treated with astrocyte derived EV‐Vector‐CR (ADEV‐Vector‐CR), astrocyte derived EV‐Vector‐IL1β (ADEV‐Vector‐IL1β), astrocyte derived EV‐CK1‐CR (ADEV‐CK1‐CR), and astrocyte derived EV‐CK1‐IL1β (ADEV‐CK1‐IL1β) for 18h. **(a‐c)** Orthogonal views of Z‐stack images show **(a)** GSK3 and Myc‐CK1, **(b)** APC and Myc‐CK1, **(c)** Axin1 and Myc‐CK1 protein co‐localization. Data are mean ± SEM, *n* = 6. ** = *p *< 0.01, One‐way ANOVA with Tukey post hoc comparisons. **(d)** β‐catenin expression was measured by western blot. *n* = 3. ** = *p *< 0.01, One‐way ANOVA with Tukey post hoc comparisons. **(e)** Neurons were treated with astrocyte derived EV‐CR (ADEV‐CR), astrocyte derived EV‐IL1β (ADEV‐IL‐1β), astrocyte derived EV‐siCK1‐IL1β (ADEV‐siCK1‐IL‐1β), and Wnt agonist (20μM) for 18h. The binding of β‐catenin to *hnrnpc* promotor sites at ‐2.3 kb, ‐2.1 kb, ‐0.9 kb, and transcription start site (TSS) were measured by chromatin immunoprecipitation ChIP‐qPCR analysis. Data are mean ± SEM of *n* = 3‐8 independent experiments per condition. One‐way ANOVA with Tukey post hoc comparisons.
**Figure S5 (relate to Figure 7). Nanoparticles tracking analysis of GLAST‐1^+^ EVs. (a)** Normal individuals’ and AD patients’ astrocytes derived extracellular vesicles (GLAST‐1^+^ EVs) were isolated from plasma. The GLAST‐1^+^ EVs were measured by nanoparticles tracking systems (NanoSight). N = 4.
