# Supplementary material for: Astrocytes deliver CK1 to neurons via extracellular vesicles in response to inflammation promoting the translation and amyloidogenic processing of APP
Source: J Extracell Vesicles. 2020 Dec 31;10(2):e12035. doi: 10.1002/jev2.12035 (PMC7775567; doi:10.1002/jev2.12035)
Supplement: Supplementary file 1 — Table S1. Primers used for RT‐qPCR This table showing the primers sequences used in this study Table S2. Primers used for ChIP‐qPCR This table showing the primers sequences used for ChIP experiment. Table S3. Antibody used in this study WB: Western Blot; IF: Immunofluorescence; IP: immunoprecipitation; ChIP: Chromatin immunoprecipitation Table graphs showing the information of Alzheimer's Disease patients’ age, gender, clinical stage, and concentration of CSF Aβ, tau, p‐tau as well as the age and gender matched normal individuals. Figure S1 (relate to Figure 2) Astrocyte derived EV‐IL‐1β induced neuronal APP C terminal fragmentation. (a) Astrocyte derived extracellular vesicles shed in response to IL‐1β (200 ng/ml; astrocyte derived EV‐IL1β as ADEV‐IL‐1β) or CR (astrocyte derived EV‐CR as ADEV‐CR) were isolated from media using a multi‐step ultracentrifugation. Representative Western Blot of IL‐1β is shown for the indicated treatment conditions. Recombinant IL‐1β (0.2μg) was included as a positive control. Data are mean ± SEM of n = 3 independent experiments per condition. NS = no significant changes, One‐way ANOVA with Tukey post‐hoc comparisons. (b) Neurons were treated with astrocyte derived EV‐CR (ADEV‐CR) and astrocyte derived EV‐IL‐1β (ADEV‐IL‐1β) for 24h. Full‐length (FL‐APP) and C‐Terminal Fragments (APP‐CTFs) of APP were detected by Western Blot using APP (Y188) antibody. Data are mean ± SEM of n = 3 independent experiments per condition. * = p < 0.01, One‐way ANOVA with Tukey post‐hoc comparisons. Figure S2 (relate to Figure 3). IL‐1β did not modulate APP and Aβ1‐42 expression. (a) 200 ng/ml recombinant IL‐1β treated primary neurons for 24h. The APP protein expression were detected by western blot. (b) representative immunofluorescent images of primary neurons showing APP (blue), BACE1 (green), GM1 (red). Merged images show the co‐localization of APP, BACE1 and GM1 as white. Bar graph shows the quantitation of percent co‐localized area along dendritic bran [file JEV2-10-e12035-s001.docx]

**Supplementary Figures**

**
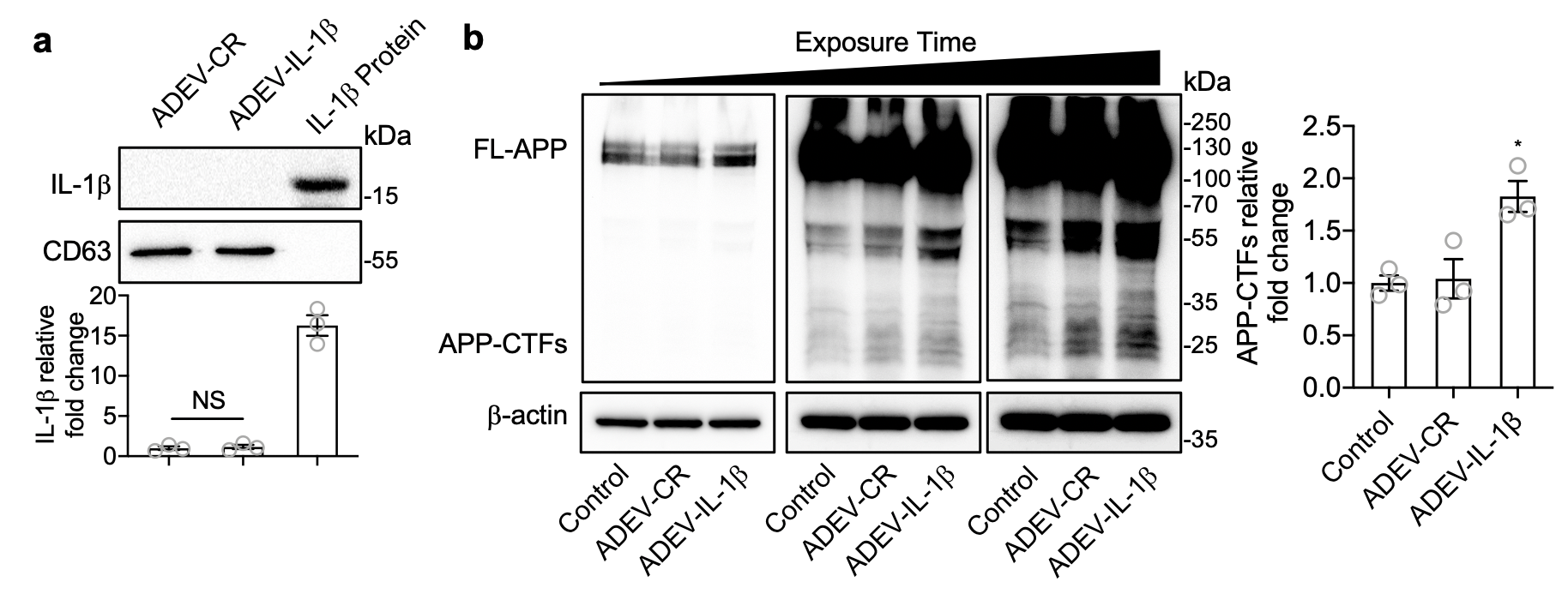
**

**Figure S1 (relate to Figure 2) Astrocyte derived EV-IL-1β induced neuronal APP C terminal fragmentation. (a)** Astrocyte derived extracellular vesicles shed in response to IL-1β (200ng/ml; astrocyte derived EV-IL1β as ADEV-IL-1β) or constitutively released (astrocyte derived EV-CR as ADEV-CR) were isolated from media using a multi-step ultracentrifugation. Representative Western Blot of IL-1β is shown for the indicated treatment conditions. Recombinant IL-1β (0.2μg) was included as a positive control. Data are mean ± SEM of n=3 independent experiments per condition. NS=no significant changes, One-way ANOVA with Tukey post-hoc comparisons. **(b)** Neurons were treated with astrocyte derived EV-CR (ADEV-CR) and astrocyte derived EV-IL-1β (ADEV-IL-1β) for 24h. Full-length (FL-APP) and C-Terminal Fragments (APP-CTFs) of APP were detected by Western Blot using APP (Y188) antibody. Data are mean ± SEM of n=3 independent experiments per condition. *=p<0.01, One-way ANOVA with Tukey post-hoc comparisons.


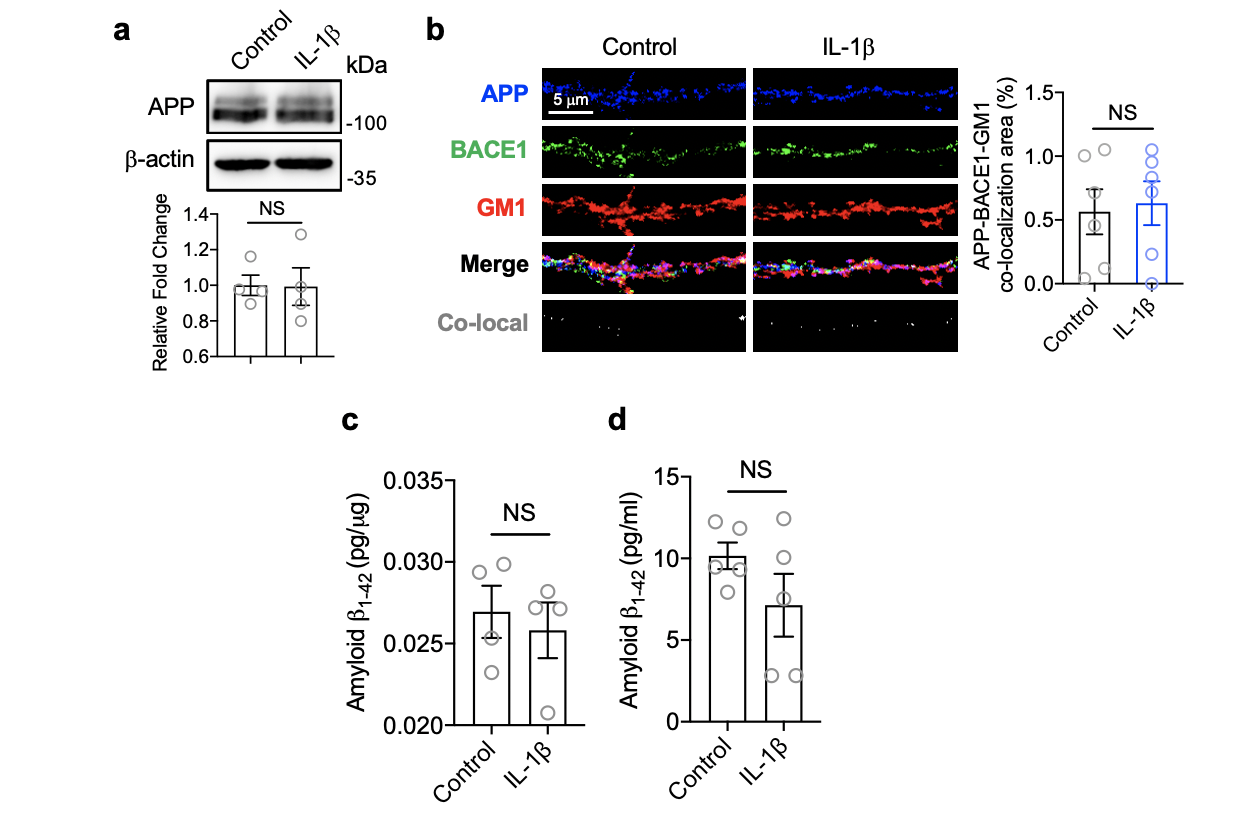


**Figure S2 (relate to Figure 3). IL-1β did not modulate APP and Aβ_1-42_ expression. (a)** 200ng/ml recombinant IL-1β treated primary neurons for 24h. The APP protein expression were detected by western blot. **(b)** representative immunofluorescent images of primary neurons showing APP (blue), BACE1 (green), GM1 (red). Merged images show the co-localization of APP, BACE1 and GM1 as white. Bar graph shows the quantitation of percent co-localized area along dendritic branches. Data are mean ± SEM of n=6. NS=no significant changes, One-way ANOVA with Tukey post-hoc comparisons. **(c-d)** 200ng/ml IL-1β treated differentiated SHSY5Y cells for 24h, the whole cell lysate **(b)** and supernatant **(c)** of Aβ_1-42_ were measured by human Aβ_1-42_ ELISA. The graphs show mean ± SEM, n=4-5. NS=no significant changes. Student’s t-test.


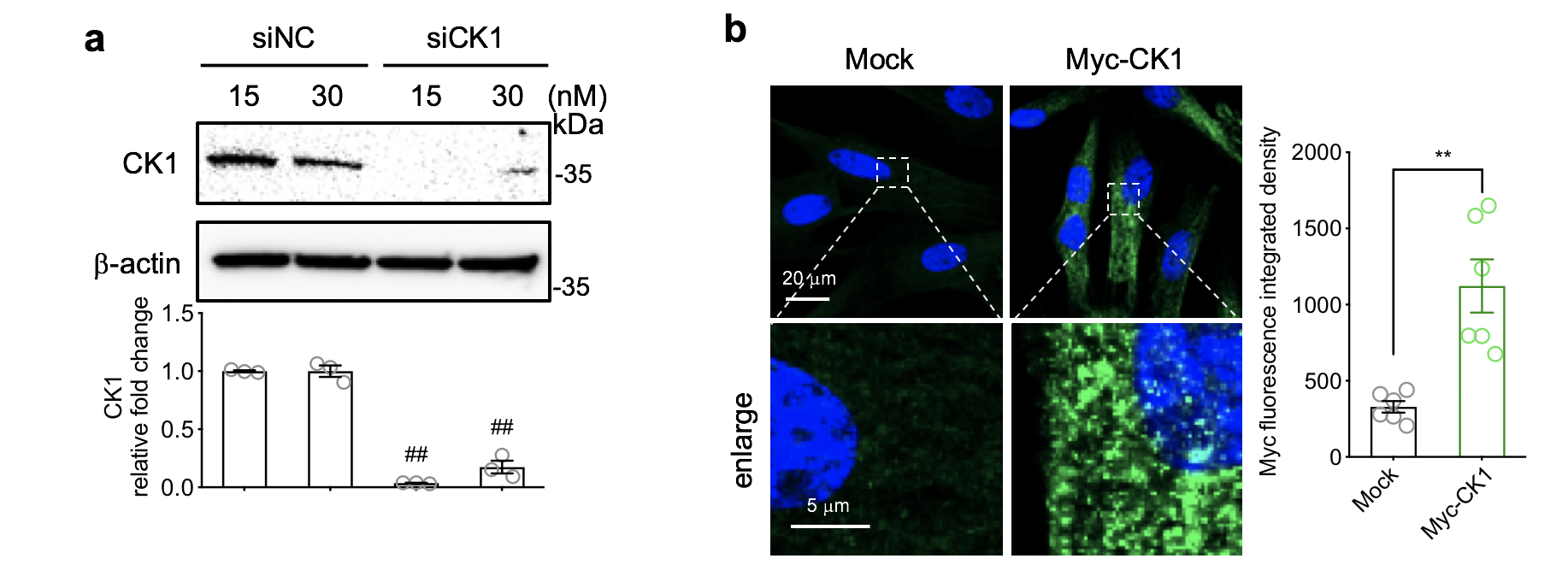


**Figure S3 (relate to Figure 4 and Figure 5). Knockdown or overexpression of CK1 in astrocytes. (a)** 15 or 30 nM of siNC and siCK1 transfected into astrocytes for 48h, the CK1 expression on astrocytes were measured by western blot. The graph bars show mean ± SEM, n=3. ^##^=*p*<0.01. One-way ANOVA with Tukey post hoc comparisons. **(b)** Astrocytes transfected with empty liposome or Myc-tagged CK1 plasmid for 48 h, the expression of Myc-CK1 were stained by immunofluorescences using Myc-tagged antibody. The graphs show mean ± SEM, n=6. **=*p* < 0.01, Student’s t-test.


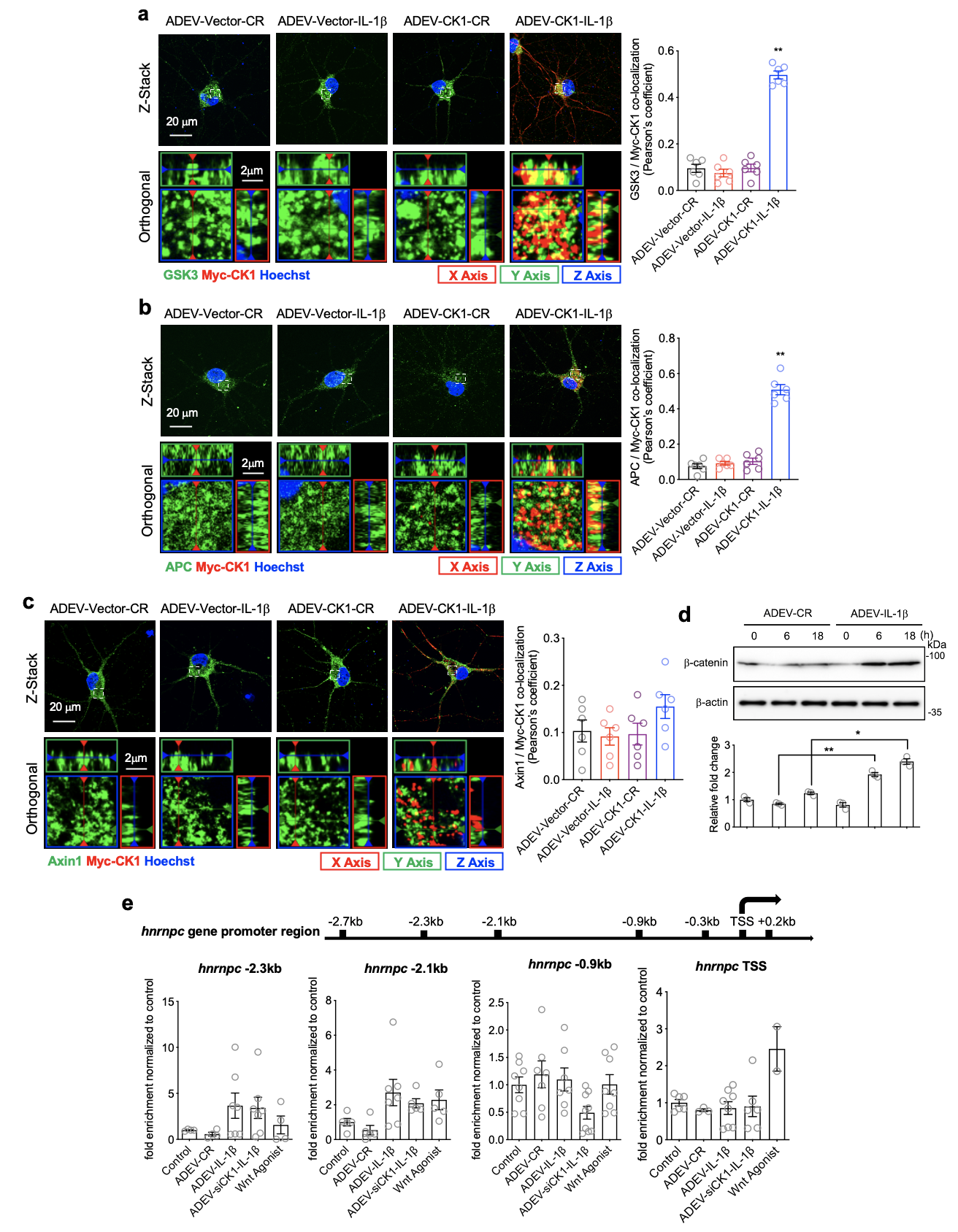


**Figure S4 (relate to Figure 6). CK1 carried in astrocyte derived EV-IL1β directly binds GSK3a/b and APC in target neurons. (a-d)** Neurons were treated with astrocyte derived EV-Vector-CR (ADEV-Vector-CR), astrocyte derived EV-Vector-IL1β (ADEV-Vector-IL1β), astrocyte derived EV-CK1-CR (ADEV-CK1-CR), and astrocyte derived EV-CK1-IL1β (ADEV-CK1-IL1β) for 18h. **(a-c)** Orthogonal views of Z-stack images show **(a)** GSK3 and Myc-CK1, **(b)** APC and Myc-CK1, **(c)** Axin1 and Myc-CK1 protein co-localization. Data are mean ± SEM, n=6. **=*p*<0.01, One-way ANOVA with Tukey post hoc comparisons. **(d)** β-catenin expression was measured by western blot. n=3. **=*p*<0.01, One-way ANOVA with Tukey post hoc comparisons. **(e)** Neurons were treated with astrocyte derived EV-CR (ADEV-CR), astrocyte derived EV-IL1β (ADEV-IL-1β), astrocyte derived EV-siCK1-IL1β (ADEV-siCK1-IL-1β), and Wnt agonist (20μM) for 18h. The binding of β-catenin to *hnrnpc* promotor sites at -2.3kb, -2.1kb, -0.9kb, and transcription start site (TSS) were measured by chromatin immunoprecipitation ChIP-qPCR analysis. Data are mean ± SEM of n=3-8 independent experiments per condition. One-way ANOVA with Tukey post hoc comparisons.


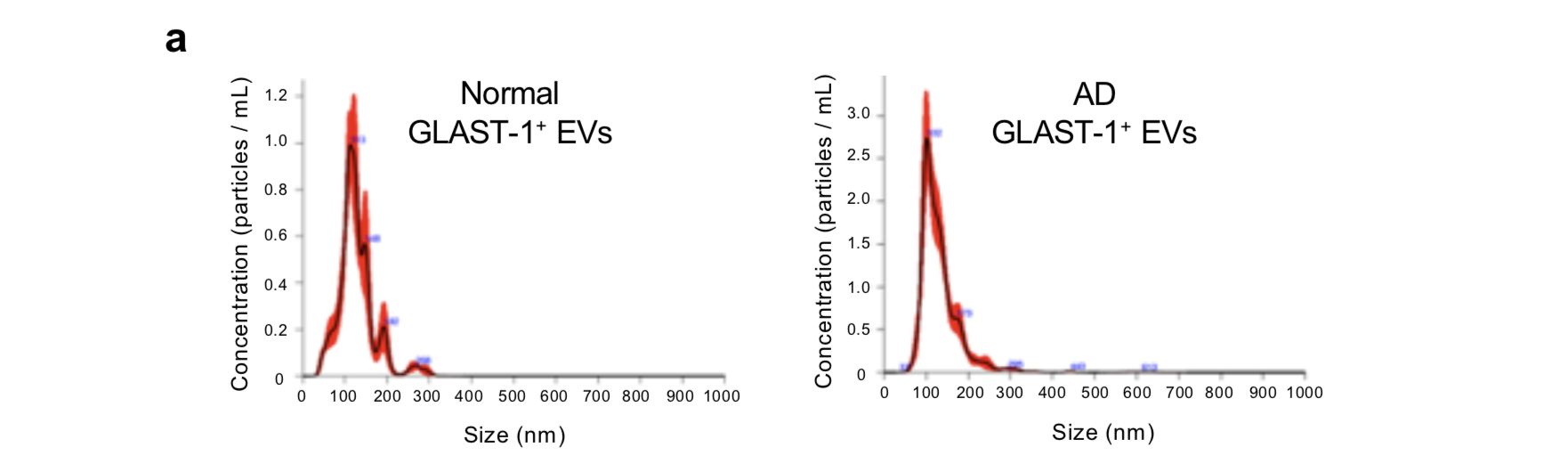


**Figure S5 (relate to Figure 7). Nanoparticles tracking analysis of GLAST-1^+^ EVs. (a)** Normal individuals’ and AD patients’ astrocytes derived extracellular vesicles (GLAST-1^+^ EVs) were isolated from plasma. The GLAST-1^+^ EVs were measured by nanoparticles tracking systems (NanoSight). N=4.

**Supplementary Tables**

**Table S1. Primers used for RT-qPCR**


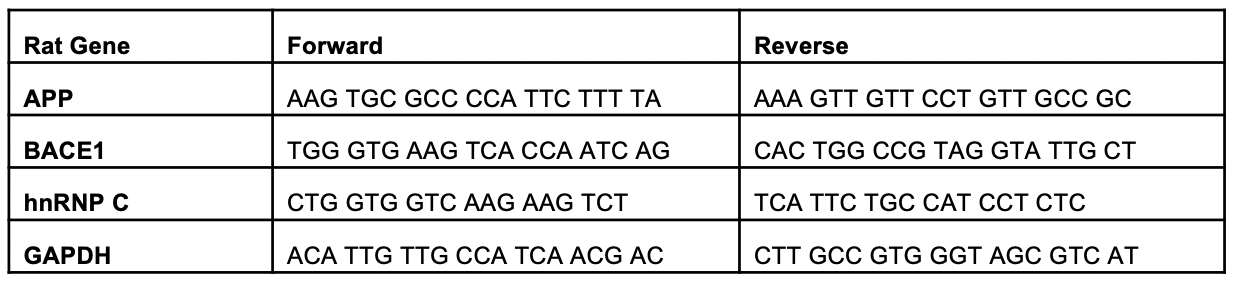


This table showing the primers sequences used in this study

**Table S2. Primers used for ChIP-qPCR**


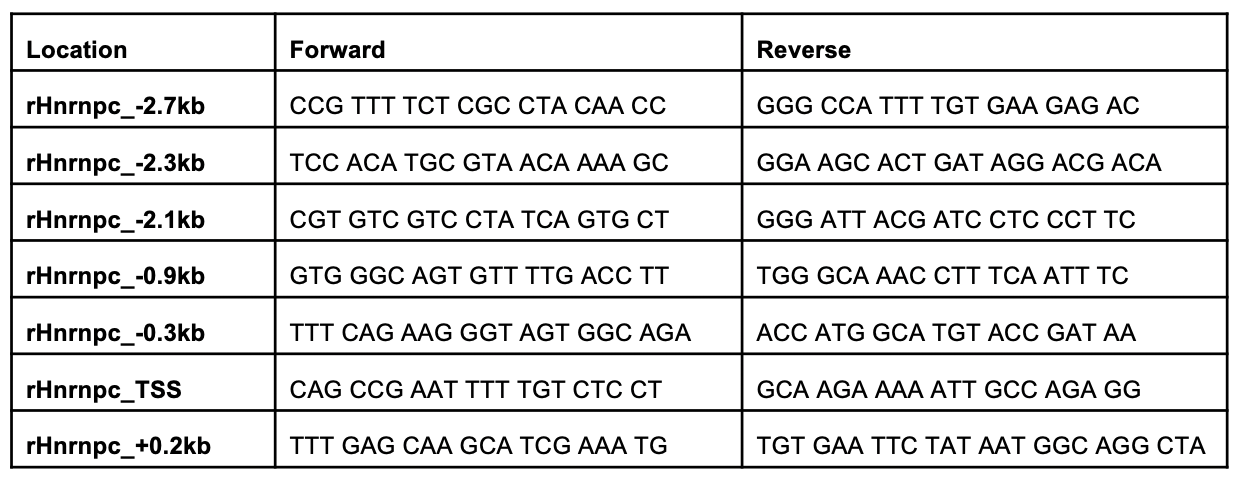


This table showing the primers sequences used for ChIP experiment.

**Table S3. Antibody used in this study**


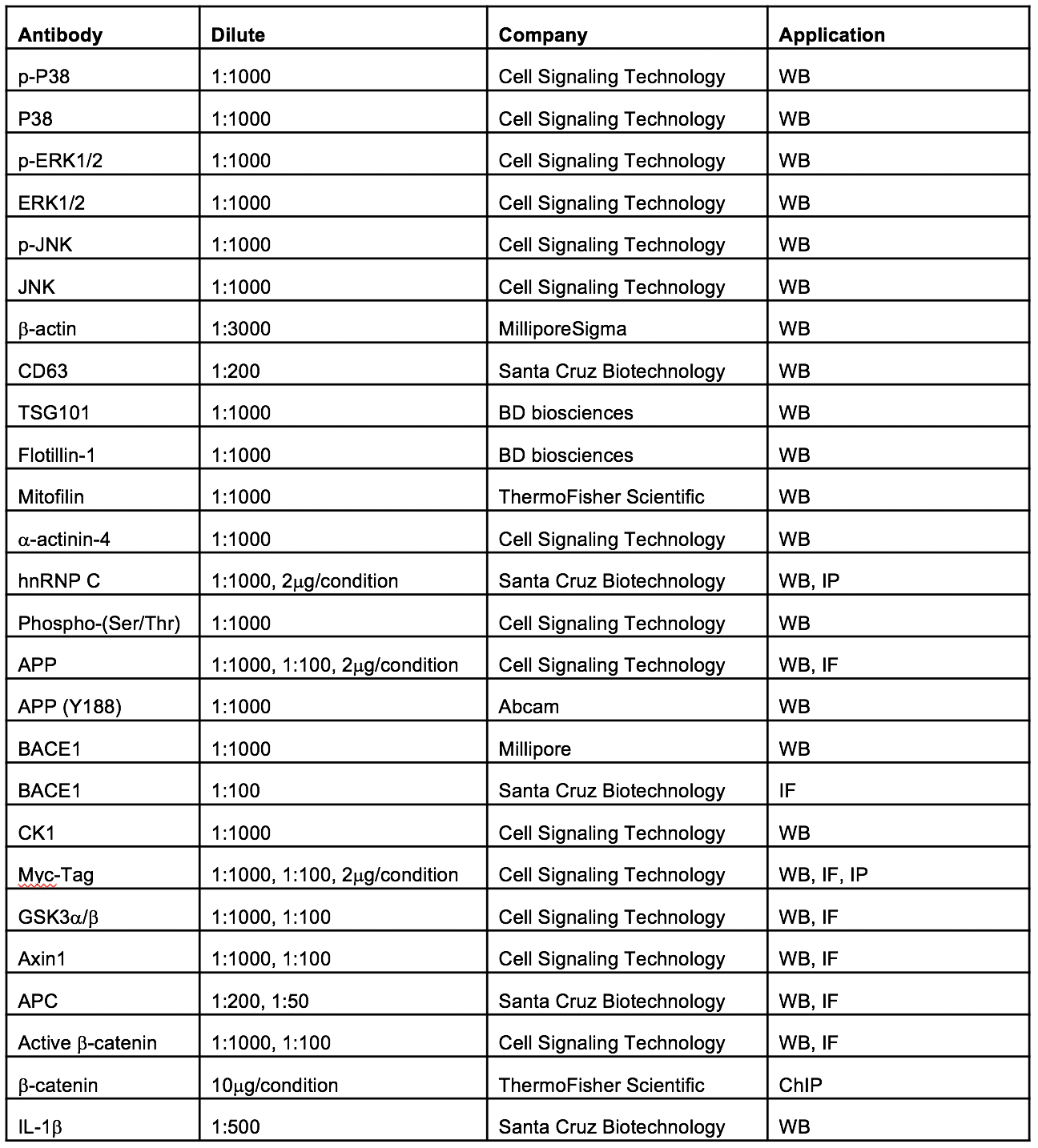


**WB: Western Blot; IF: Immunofluorescence; IP: immunoprecipitation; ChIP: Chromatin immunoprecipitation**
